# stGCL: a versatile cross-modality fusion method based on multi-modal graph contrastive learning for spatial transcriptomics

**DOI:** 10.1186/s13059-025-03896-w

**Published:** 2026-01-28

**Authors:** Na Yu, Daoliang Zhang, Wei Zhang, Zhiping Liu, Xu Qiao, Chuanyuan Wang, Miaoqing Zhao, Weiming Yue, Wei Li, Yang De Marinis, Rui Gao

**Affiliations:** 1 School of Control Science and Engineering, Shandong University, Jinan, Shandong China; 2 School of Life Sciences, Westlake University, Hangzhou, Zhejiang China; 3 Institute of Science and Technology for Brain-Inspired Intelligence, MOE Key Laboratory of Computational Neuroscience and Brain-Inspired Intelligence, MOE Frontiers Center for Brain Science, Fudan University, Shanghai, China; 4 Department of Pathology, Shandong Cancer Hospital and Institute, Shandong First Medical University and Shandong Academy of Medical Sciences, Jinan, Shandong China; 5 Department of Thoracic Surgery, Qilu Hospital of Shandong University, Jinan, Shandong China; 6 Department of Clinical Sciences, Lund University, Malmö, Sweden; 7 Oxford Centre for Diabetes, Endocrinology and Metabolism, Radcliffe Department of Medicine, University of Oxford, Churchill Hospital, Oxford, UK

## Abstract

Advances in spatial transcriptomics have enabled high-resolution mapping of tissue architecture at the molecular level, yet integrating its multi-modal data remains challenging. Here, we present stGCL, a framework for accurate and robust integration of gene expression, spatial coordinates, and histological features. stGCL employs a histology-based Vision Transformer to extract morphological features and a multi-modal graph autoencoder with contrastive learning for cross-modal fusion. In addition, we introduce a spatial coordinate correction and registration strategy to support multi-slice integration. We demonstrate that stGCL reliably identifies spatial domains, integrates vertical and horizontal tissue slices, and highlight its generalizability across platforms and resolutions.

**Supplementary Information:**

The online version contains supplementary material available at 10.1186/s13059-025-03896-w.

## Background

Deciphering the distribution of cells and their interrelationships within tissue is crucial for understanding the cellular biological functions and disease pathology [1]. Recent advances in spatial transcriptomics (ST) technologies have enabled the simultaneous capture of histological and gene expression profiles, providing spatial context that extends beyond what can be achieved with single-cell RNA sequencing (scRNA-seq) alone [2]. In situ hybridization (ISH) (e.g. seqFISH+ [3], MERFISH [4]) and in situ sequencing (ISS) (e.g. STARmap [5], 10× Xenium [6]) techniques measure the spatial distribution of mRNA transcripts with high spatial resolution. In situ capturing-based (ISC) techniques (e.g. Slide-seq [7], 10× Visium [8], Stereo-seq [9]) detect gene expression levels on a genome-wide scale at capture sites (called spots). These emerging ST technologies with new perspectives in tissue architecture, intercellular interactions, and disease mechanisms.

Analysis of ST data can elucidate anatomical structures and, reveal spatial organizations and functionalities within the milieu of complex tissues. Conventional ST data clustering methods, such as k-means and the Louvain algorithm [10], annotate gene expression information to categorize spots which are clustered into distinct domains. Methods such as Giotto [11] and BayesSpace [12] incorporate spatial relationships by modeling neighboring spots using Markov random fields, improving spatial coherence. SEDR [13], STAGATE [14], CCST [15], and GraphST [16] use deep auto-encoder networks to identify spatially distributed domains by combining expression profiles with spatial locations. Undoubtedly, these methods facilitate the integration of gene expression with spatial cellular distribution, thereby revealing intricate relationships between spatial gene expression patterns and tissue function across various areas. However, these methods typically emphasize local spatial contexts or gene expression profiles alone and may not fully leverage complementary multi-modal information (particularly high-resolution texture features derived from histology images), potentially limiting their performance in predicting tissue structures [17, 18]. K-means and the Louvain algorithm adopt only gene expression information and spatial information between spots is largely ignored. This may result in discontinuous spatial distributions of spots from the same category in the original spatial context. Methods such as Giotto, SEDR, CCST, and STAGATE incorporate spatial coordinate information into their algorithms. However, these methods discard the information on cell organization captured by histology images. It is worth noting that recently proposed algorithms such as stLearn [19] and DeepST [17], extract spatial neighborhood information and morphological features from histology images and use them to normalize gene expression before clustering. In addition, SpaGCN [20] learns features from color space of histology images and implements domain recognition using a graph convolutional network (GCN) that combines gene expression, spatial location, and histology. Despite these significant advancements, existing methods still have notable limitations in effectively capturing high-resolution, content-rich texture features within histology images. Furthermore, these methods do not fully exploit the potential in exploring and integrating discriminative information from various modalities, thereby restricting their ability to accurately characterize spatial patterns and provide comprehensive insights into the biological context.

In addition, ST sequencing is currently constrained by its inherent technological limitations, restricting its sample acquisition to a limited tissue region. To study the entire tissue region of interest, samples are often dissected into multiple slices either vertically or horizontally. Integrating multiple slices allows for the full utilization of cross-slice information, yielding a more comprehensive characterization of spatial organization and potentially enhancing the performance of spatial domain identification. At present, only a limited number of computational methods are able to perform integration task. SpaGCN [20] jointly analyzes multiple serial slices by creating a block adjacency matrix and concatenating gene expression matrices. However, it ignores the correction of the spatial location between slices. STAGATE [14] constructes the three-dimensional spatial neighbor network (3D SNN) to integrate multiple consecutive slices for 3D spatial domain identification. Nevertheless, STAGATE requires aligned slices as input and cannot handle horizontal integration of multiple adjacent slices. SPIRAL [21] aligns tissue slices using spatial proximity and transcriptional similarity, whereas PASTE2 [22] leverages the mean RGB values of histology images to assist alignment. The recently proposed GraphST [16] utilizes the PASTE [23] algorithm to align spatial coordinates and integrates multiple tissue slices for joint analysis. However, SPIRAL, PASTE2 and GraphST only consider 2D coordinates, restricting the accurate depiction of the 3D spatial tissue structure across multiple slices. The alignment of spatial coordinates across tissue slices remaines highly challenging because of the differences in the structure of the slices and their position on the array [23]. Therefore, effective and precise integration of spatial data, whether horizontal or vertical, is crucial but has yet to be fully achieved in ST research.

To overcome these limitations, we developed a versatile cross-modality fusion framework named stGCL based on multi-modal graph contrastive learning (GCL) for spatial domain detection, multi-slice integration, and related downstream analyses. stGCL adopts the histology-based Vision Transformer (H-ViT) to effectively encode histological features such as morphology and spatial distribution of tissue cells. By combining multi-modal graph attention auto-encoder (GATE) with contrastive learning, stGCL fuses transcriptional profiles, histological features as well as spatial information simultaneously. With the above two key steps of data processing and fusion, stGCL generates effective embeddings for accurately identifying spatially coherent regions across transcriptional and histological profiles. In addition, we introduce a novel multi-slice alignment algorithm based on tissue slice edge structure, which not only ensures the efficient integrated analysis of ST data across slices, but also uncovers spatial heterogeneity in 3D tissue structure on a broader scale. We conducted extensive experimental tests on spatial transcriptomics data obtained from various experimental platforms, demonstrating the superior performance of stGCL from multiple perspectives including domain identification, multi-slice integration and biological mechanism analysis. The performance of stGCL was further confirmed by its ability to distinguish tissue spatial heterogeneity and decipher biological functions in a bronchiolar adenoma (BA) dataset.

## Results

### Overview of stGCL

**Fig. 1:**
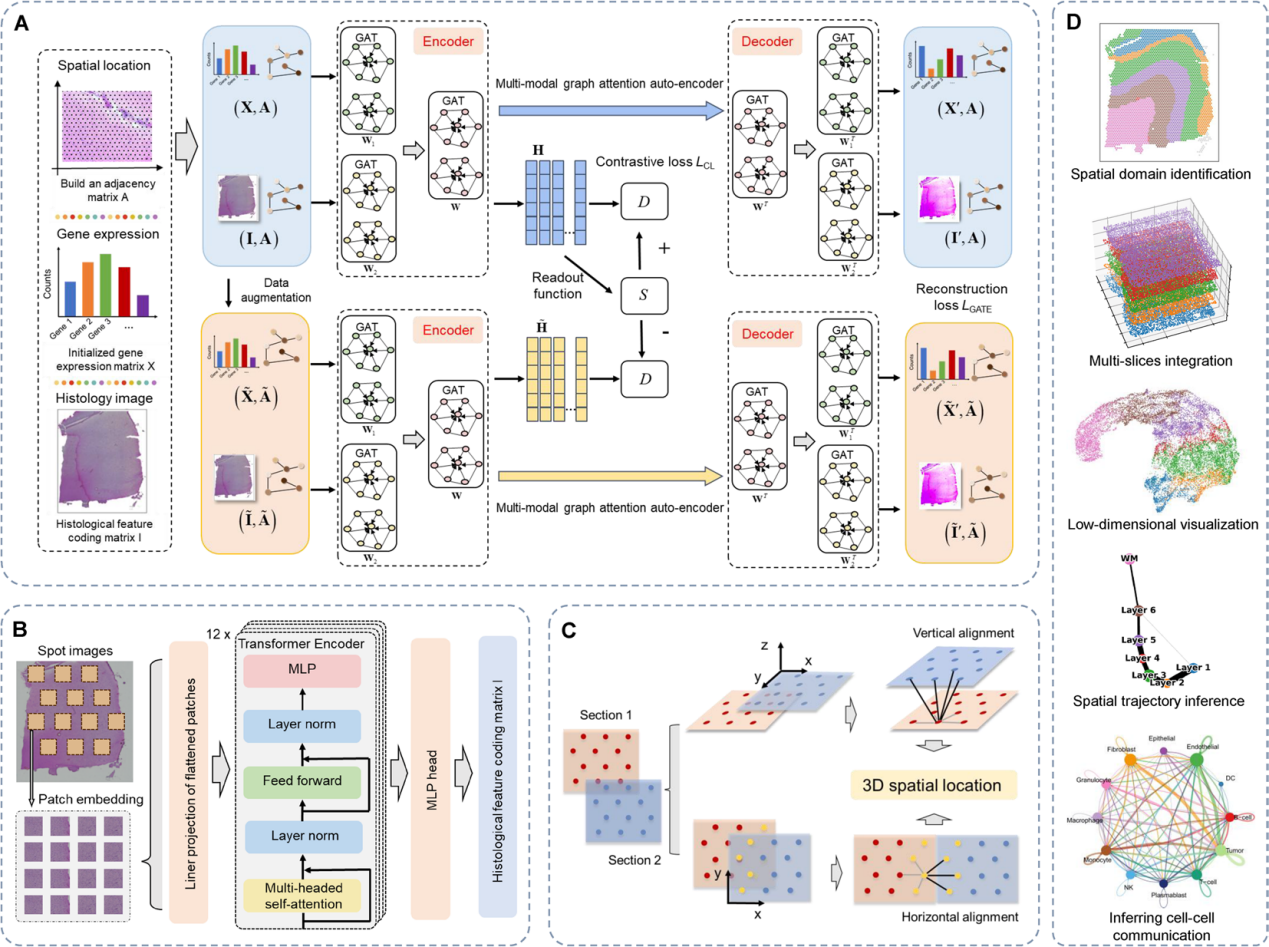
Schematic diagram of the stGCL. **A** stGCL integrates gene expression profiles, spatial information and histological profiles as input, subsequently deriving low-dimensional latent representations through multi-modal graph contrastive learning. In particular, stGCL aims to minimize reconstruction loss and contrastive loss during the training phase. This process involves the utilization of multi-modal GATE and contrastive learning techniques to produce a comprehensive spot joint embedding. **B** stGCL utilizes a modified H-ViT model for the extraction of morphological features from histology images. **C** In ST datasets with multiple tissue slices, stGCL vertically or horizontally aligns these sections along the edge of the cut surface to maintain spatial consistency. After alignment, it constructs a spatial neighborhood graph using the coordinated locations, encompassing location data from all slices. **D** The output of stGCL is adaptable for diverse downstream tasks, including spatial domain identification, trajectory inference, and cell–cell communication inference

The overall framework of stGCL is depicted in Fig. 1A. Briefly, the core algorithm of stGCL integrates takes gene expression data, spatial coordinates, as well as histology image as inputs, and generate spot embeddings that capture expression similarity, morphological similarity, and spatial proximity. These embeddings can be further used for exploring downstream analyses, such as spatial domain identification, low-dimensional visualization, trajectory inference and cell–cell communication analysis (Fig. 1D).

The stGCL adopts a modified H-ViT to efficiently encode the histological features of the spots by dividing each spot image into multiple patches, thereby obtaining the histological feature encoding matrix $\mathbf{I}$ (Fig. 1B). Next, a spatial neighborhood graph is constructed based on spatial coordinates to characterize the relationship between the spots. stGCL then uses data augmentation, a multi-modal GATE and contrastive learning to embed spot gene expression, histological information and spatial similarity to generate the low-dimension embedding $\mathbf{H}$ (Fig. 1A). Specifically, the proposed multi-modal GATE learns spot embedding by iteratively aggregating gene expression features and histological features from adjacent spots. This step encodes each spot and its corresponding negative sample (see Methods), and then decodes the embeddings to reconstruct gene expression and histological profiles. In contrastive learning, stGCL maximizes the mutual information between the node representation and the global information of the entire graph, thereby endowing the spot embedding with both local and global structural patterns. Finally, stGCL combines reconstruction loss and contrastive loss to update the spot embedding $\mathbf{H}$ to make it more informative and discriminative.

In addition to processing individual slices, stGCL also possesses embedding capabilities for horizontal and vertical multi-slice data (Fig. 1C). The spatial coordinates of multiple slices are first aligned vertically or horizontally using a novel alignment strategy, and a spatial neighborhood network is built (see Methods). Then stGCL integrates gene expression data, histological features and spatial information from multiple slices to learn spot embedding with more comprehensive cross-slice information, thereby encouraging the smoothing of adjacent spot features within and across slices as well as mitigating the batch effect. Finally, spot embedding can be applied for downstream analytical tasks.

### stGCL accurately dissects tissue structures from the dorsolateral prefrontal cortex dataset

**Fig. 2:**
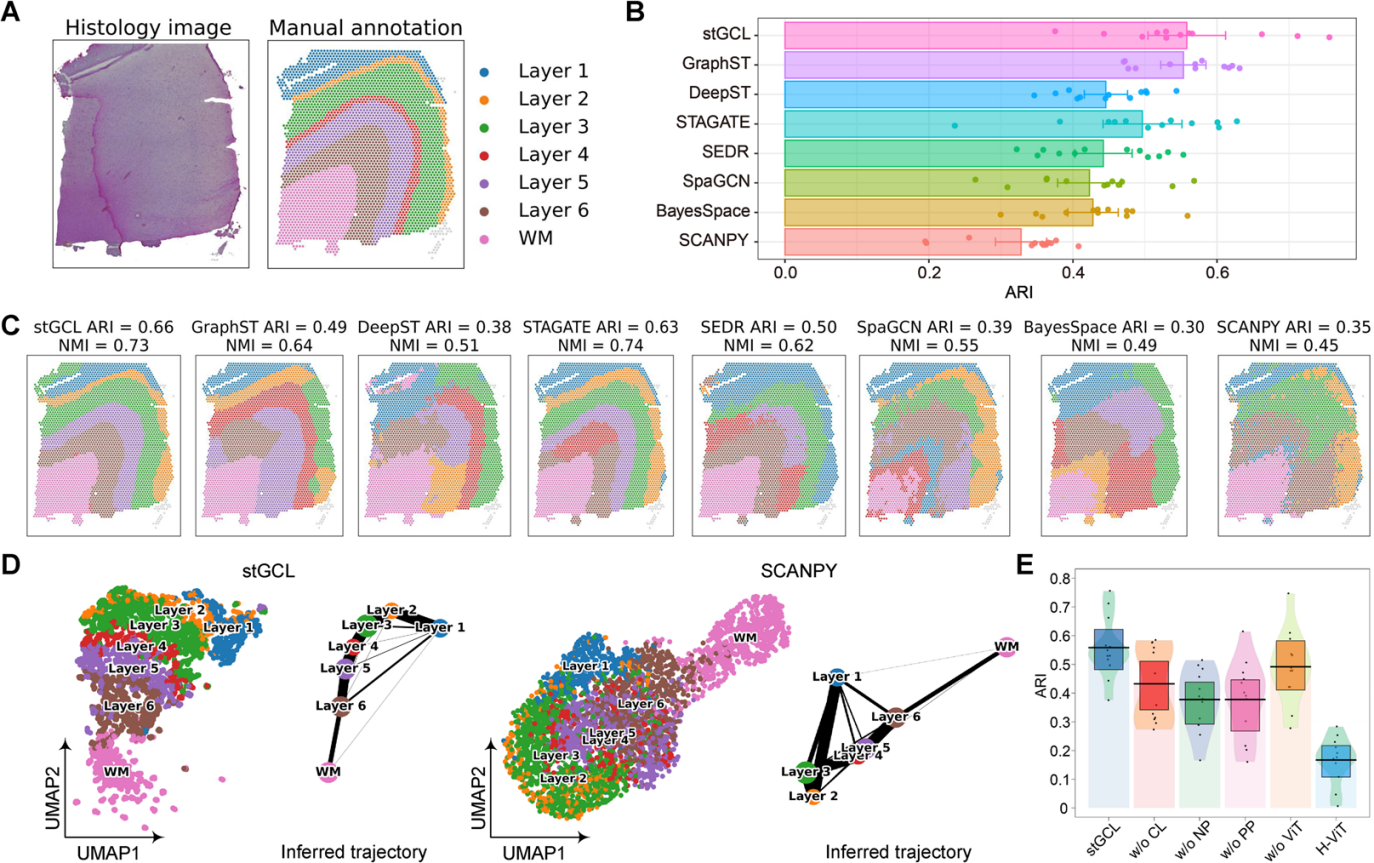
stGCL enables accurate identification of layer structures in the DLPFC data. **A** Hematoxylin and eosin (H&E) staining image and manually annotated tissue structures for DLPFC slice 151,674. **B** Clustering results of eight methods on 12 DLPFC slices. The results are evaluated by ARI scores. **C** Spatial domains identified by eight methods within the DLPFC slice 151,674. **D** UMAP visualizations and PAGA graphs for DLPFC slice 151,674 from the embeddings of stGCL and SCANPY. **E** The ARI pirate graph of ablation studies for stGCL. The ARI scores of each slice is represented by points. Horizontal lines represent the mean scores for each condition and the boxes denote the 95% confidence intervals

To evaluate the performance of stGCL, we applied stGCL to 12 human dorsolateral prefrontal cortex (DLPFC) slices generated by the 10× Visium platform [24] for spatial domain detection task. Based on cytoarchitecture and gene markers, the DLPFC dataset was manually annotated into neuronal layers (Layer 1-Layer 6) and white matter (WM) (Fig. 2A), which serve as ground truth. For performance comparison, we benchmarked stGCL against other methods, including the non-spatial clustering approach (SCANPY [25]) as well as six recently proposed spatial clustering methods (BayesSpace [12], SpaGCN [20], SEDR [13], STAGATE [14], DeepST [17], and GraphST [16]). These methods were applied to the DLPFC dataset using their recommended default parameters, allowing for a comprehensive and fair assessment of stGCL's capabilities in relation to current methodologies.

In the analysis of 12 slices, stGCL demonstrated superior efficiency in identifying brain tissue structures, achieving the highest level of clustering performance (mean ARI = 0.56, mean NMI = 0.65) (Fig. 2B and Additional file 1: Fig. S1). It is noteworthy that clustering methods based on spatial information significantly outperform those not utilizing spatial data (Fig. 2B, C). This indicates that considering spatial neighborhood information is crucial for the accurate identification of functional regions in tissues. Additionally, we note that despite both SpaGCN and DeepST incorporating histological information, their performance falls short compared to SEDR, STAGATE, GraphST, and stGCL. This phenomenon is likely attributable to the suboptimal integration strategies of histological information adopted by these methods. Unlike other approaches, stGCL treats histology images as an independent data modality and employs a contrastive learning method for data fusion. This fusion technique enables the full utilization of information from multiple modalities, which is fundamental to stGCL's superior performance compared to other methods. For instance, in the analysis of slice 151,674, which comprises 3,673 spots and 19,856 genes, the clustering results obtained by stGCL exhibit the clearest and most continuous cortical layer boundaries (ARI = 0.66, NMI = 0.73). Simultaneously, stGCL and STAGATE are the only methods that can accurately depict Layer 2 (Fig. 2C). However, stGCL identifies Layer 2 as a thin layer, most closely matching with the shape of the annotated cortical layer.

To better demonstrate the effectiveness of the latent information discovered by stGCL, we conducted further developmental trajectory inference analysis on slice 151,674 (Fig. 2D). In brief, utilizing the latent embedding information obtained from stGCL, we employ uniform manifold approximation and projection (UMAP) to reveal distances between spatial domains and perform trajectory inference using the partition-based graph abstraction (PAGA) [26] algorithm. As shown in Fig. 2D, the individual cortical layers are well organized in the UMAP plot generated by stGCL. Additionally, we used SCANPY to plot a corresponding UMAP diagram for comparison. In the SCANPY UMAP plot, only the Layer 1 and WM show effective separation, while spots of other layers exhibit substantial mixing. In contrast, the PAGA results derived from the latent information of stGCL distinctly display different layers. Moreover, the PAGA results show a near-linear developmental trajectory from Layer 1 to Layer 6, with greater similarity observed between adjacent cortical layers.

To further test the effectiveness of the core modules of stGCL, ablation experiments were conducted on 12 DLPFC slices. As shown in Fig. 2E**,** the performance of stGCL decreases by 6.7% when histological information is disregarded (w/o H-ViT). The mean ARI score for encoding histological image features using H-ViT alone is 0.17. This demonstrates that the H-ViT module, which considers histological images, effectively extracts histological information and is clearly necessary for enhancing the performance in domain identification. Additionally, we found that the performance of stGCL is affected by factors such as the absence of contrastive learning (CL), positive and negative pairs (PP and NP) in CL. When stGCL utilizes all modules and achieves feature fusion, it is able to achieve optimal performance.

### stGCL improves integration of multiple tissue slices

The study of gene expression in relation to spatial context often necessitates the integration of multiple tissue slices to construct a more detailed and comprehensive gene expression atlas. To conduct spatial transcriptomic studies on tissue regions of interest, experimental samples often need to be divided into multiple slices for sequencing, both horizontally and vertically. Consequently, to obtain more complete and continuous spatial transcriptomic information of tissues or organs, computational methods for horizontal or vertical stitching and alignment are now essential [16]. However, most current analysis methods are only applicable to individual tissue slices and cannot jointly identify spatial domains from multiple slices, either horizontally or vertically. Furthermore, batch correction methods developed for scRNA-seq only consider gene expression relationships and neglect spatial information, making batch effects difficult to resolve. Therefore, joint modeling and analysis of multiple slices represent a significant challenge in the field of spatial transcriptomics.

**Fig. 3:**
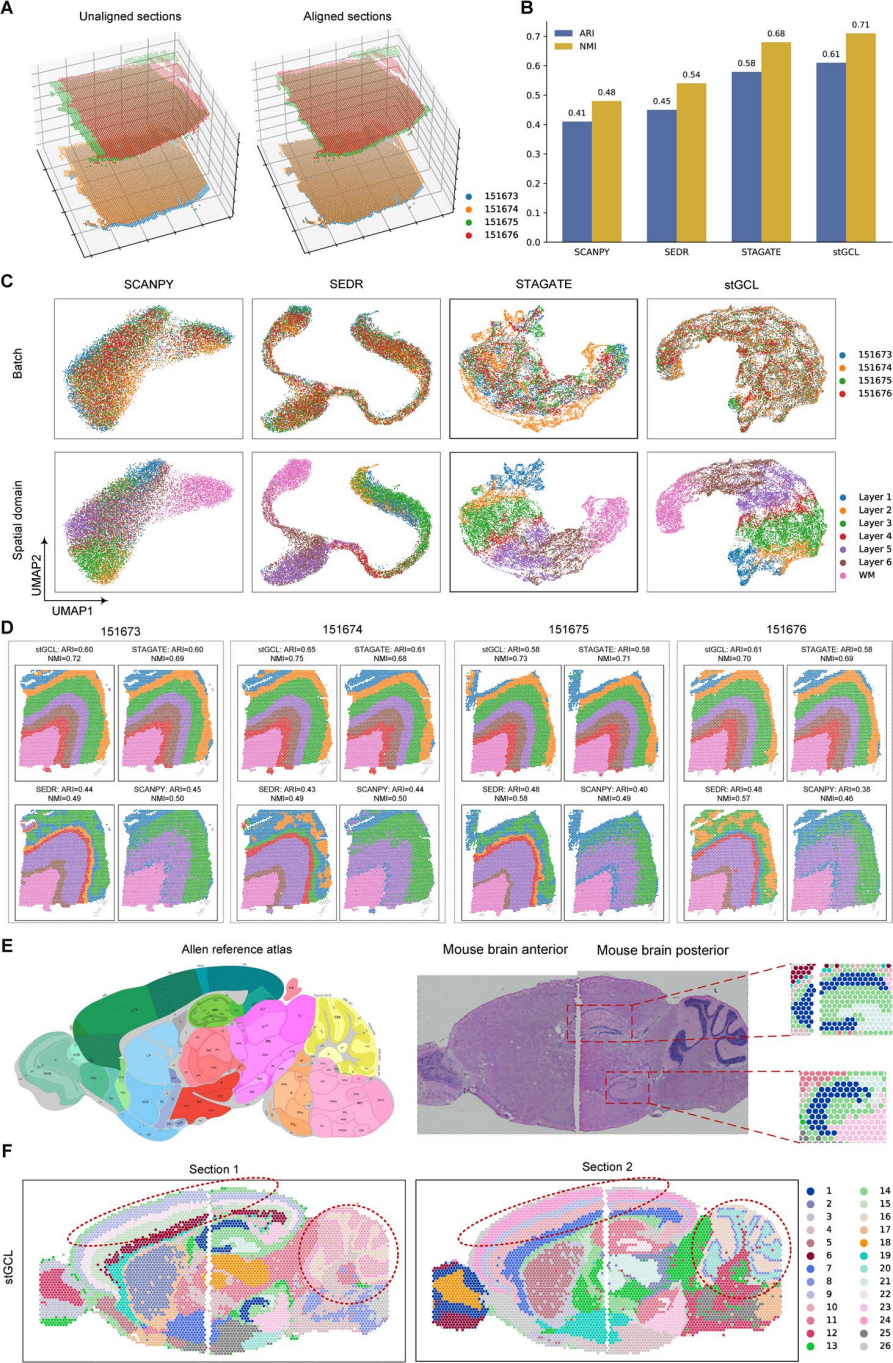
stGCL improves both vertical and horizontal integration within DLPFC datasets, as well as in anterior and posterior sagittal slices of the mouse brain. **A** Visualization of four DLPFC slices in 3D space (left). The aligned results from stGCL are shown in right panel. **B** A histogram depicting the results of spatial clustering for four DLPFC slices is shown, executed through four different methods. **C.** The UMAP visualization showcases an integrated analysis of four slices utilizing SCANPY, SEDR, STAGATE, and stGCL. In this representation, spots are color-coded to represent different slices (top) and cortical layers (bottom), respectively. **D** Identification of spatial domains in four tissue slices using four different integration algorithms. **E** Anatomical annotations from the Allen Mouse Brain Atlas (left) and H&E images of the mouse brain sagittal anterior and posterior (right). The red boxes indicate the dorsal and ventral horns of the hippocampus region identified by stGCL in Sect. 1, which both contain the cornu ammonis (CA) and dentate gyrus (DG) structures. **F** Results of horizontal integration of stGCL on two mouse brain sections. Red circles mark the cerebral cortex (CTX) and cerebellum (CBX) regions identified by stGCL, respectively

stGCL employs a novel multi-slice alignment algorithm based on tissue slice edge structure to address this challenge. It explores the neighborhood information within each slice and between adjacent slices across batches through spatial alignment of multiple tissue slices. To validate the efficacy of stGCL, we applied it to four consecutive slices from the aforementioned benchmark DLPFC dataset (151,673, 151,674, 151,675, and 151,676). The first two slices and the last two slices are directly adjacent, and the middle two slices are 300 μm apart [24]. stGCL successfully aligns the four slices in 3D space (Fig. 3A). In Fig. 3C, it is evident that SCANPY and SEDR mix the four slices together, failing to distinctly differentiate the cortical layers. While STAGATE employs a 3D spatial neighbor network (3D SNN), its results still exhibit batch-specific separations of specific slices. stGCL effectively performs low-dimensional embedding of the multi-slices, producing clear and ordered separations between the different layers (from Layer 1 to Layer 6 and WM). This demonstrates that stGCL not only corrects batch effects but also preserves tissue heterogeneity information. The outstanding performance of stGCL can be attributed to the fact that the cross-slice spatial neighborhood graph alleviates the feature distribution differences between batches and the spot representation learned by CL captures the global features of tissue slices. Moreover, stGCL achieved the highest ARI score (ARI = 0.61, NMI = 0.71) for the integration of the four slices (Fig. 3B). Notably, the integrated analysis using four tissue slices with stGCL demonstrated higher clustering accuracy than the analysis using only one slice (Fig. 3D and Additional file 1: Fig. S1). For instance, stGCL not only captured smooth domain boundaries but also depicted the WM structure more closely matching the expected truth. This indicates that integrating multiple tissue slices facilitates the consideration of cross-slice information, thereby enhancing the performance of spatial domain identification. Additionally, we conducted a vertical alignment analysis of 12 DLPFC slices using stGCL. Here again, stGCL obtained the highest ARI and NMI scores and exhibited superior integration performance (Additional file 1: Fig. S2 A-C). In summary, stGCL is able to robustly integrate multiple slices generated on the same platform to produce coherent 3D reconstructions.

Next, we applied stGCL to sagittal anterior slices (Sect. 1 and Sect. 2) of mouse brain [16], which exhibited evident batch effects. We vertically aligned Sect. 1 and Sect. 2 in 3D space (Additional file 1: Fig. S2F) and the UMAP plots were then used to visually evaluate the integration results (Additional file 1: Fig. S2G). stGCL successfully integrated these two slices, achieving an effective low-dimensional spatial representation (Additional file 1: Fig. S2F, G). This demonstrates stGCL's ability to efficiently integrate tissue slices from multiple batches and accurately align tissue structures across different slices.

In response to the issue of limited size in spatial transcriptomics capture regions, we also tested the horizontal multi-slice integration capability of stGCL. Here, we adopted two sections of mouse brain data [16], where both sections were divided into sagittal anterior and posterior slices. For a fair qualitative comparison, the number of clusters in stGCL was set to 26, the same as in the previous studies SpaGCN [20] and GraphST [16]. Compared to SpaGCN, the tissue structures represented by stGCL and GraphST align more closely with annotations in the Allen Reference Atlas [27] (Fig. 3E), while SpaGCN lacks clear spatial separation and exhibits many noisy spots (Fig. 3F and Additional file 1: Fig. S3). stGCL and GraphST align along the shared edges of the anterior and posterior brain, better reflecting the spatial adjacency of the two slices. Furthermore, stGCL outperforms GraphST in capturing complex and fine tissue structures. Specifically, the cerebral cortex (CTX) and cerebellum (CBX) regions delineated by stGCL are more consistent with annotations from the anatomy. In addition, stGCL clearly localizes the dorsal and ventral horns of the hippocampus region, and accurately captures the "cord-like" and "arrow-like" structures (cornu ammonis (CA) and dentate gyrus (DG) domains) within them (Fig. 3E, F). Overall, stGCL enables efficient identification of shared layer structures across tissue slices through horizontal integration.

We further explored the biological functionality of the spatial domains identified by stGCL by examining on the identified marker genes (Supplementary Fig. S4). *Neurod6*, a gene encoding a neural transcription factor, is highly expressed in the CA region of the hippocampus [28]. *C1ql2*, a known biomarker for the DG, is enriched in the DG region [29]. *Gng4* shows differential expression in the granule layer of the main olfactory bulb (MOBgr) [30, 31]. *Prkcd* and *Lamp5* are respectively enriched in the thalamus and superficial cortex layer [32, 33]. These gene expression variations, confirmed in previous studies, are validated in the results of stGCL (Additional file 1: Fig. S4). Therefore, these findings underscore the significance of stGCL in accurately identifying domains across tissue slices and successfully revealing spatially specific gene expression patterns.

### stGCL demonstrates robustness and scalability on spatial transcriptomic data across platforms and resolutions

The analysis algorithms for spatial transcriptomics data exhibit varying sensitivity to different experimental platforms [34]. This sensitivity arises primarily from variations in sample processing, data acquisition methods, and the quality and characteristics of data across different platforms. For instance, different platforms may vary in terms of spatial resolution, gene detection sensitivity, and the complexity of sample processing. To test the universality of stGCL, we conducted an analysis and comparison on spatial transcriptomics data obtained from different platforms with varying spatial resolutions and modalities.

**Fig. 4:**
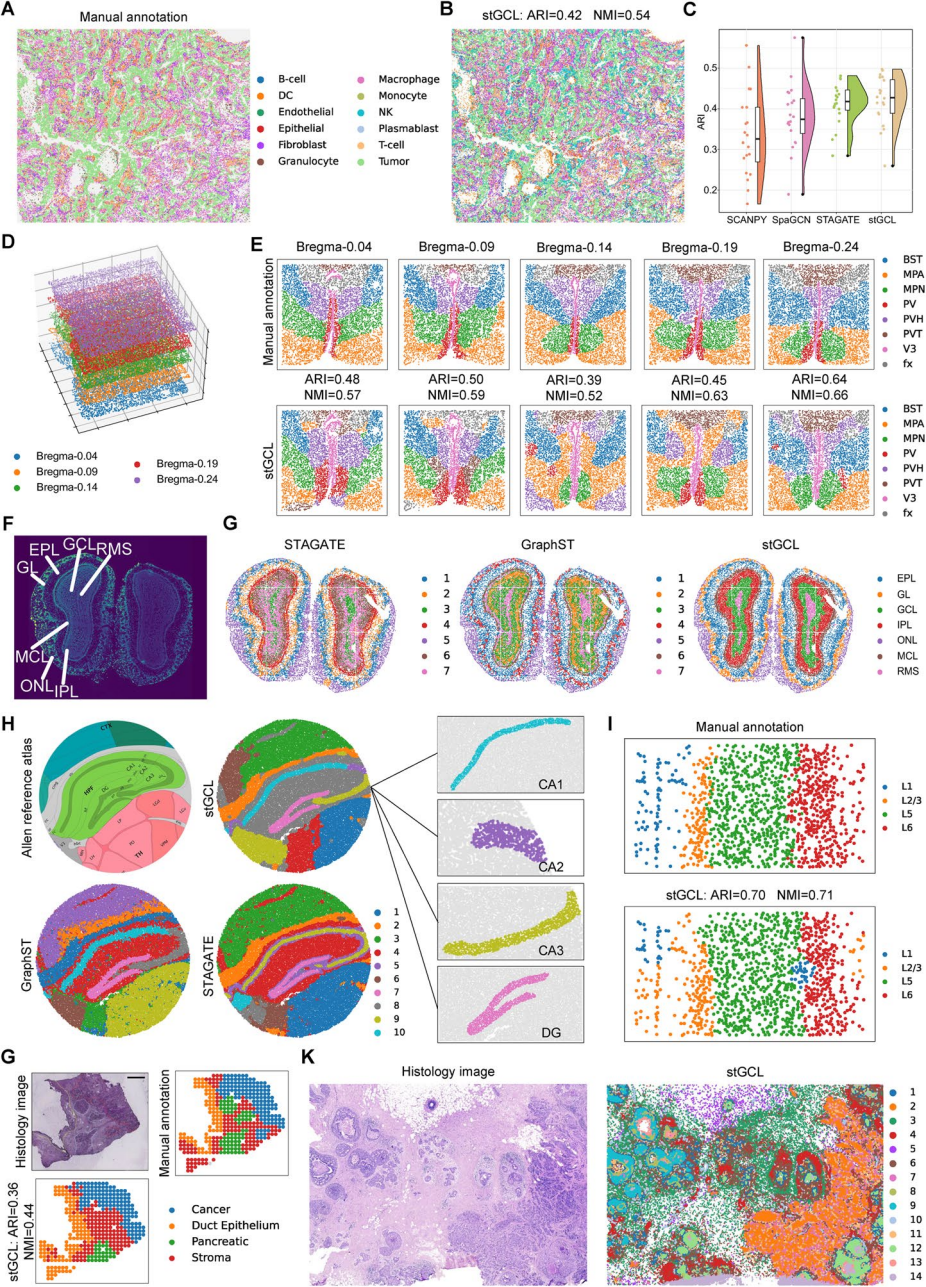
stGCL achieves superior clustering performance on datasets profiled by different ST platforms. **A** Manual annotation of human NSCLC NanoString CosMx SMI data containing 20 FOVs. **B** Visualization of the clustering results of stGCL. **C.** Raincloud plot of ARI scores for four methods at 20 FOVs of NSCLC data. **D** stGCL aligns five consecutive hypothalamic preoptic area slices (Bregma-0.04, Bregma-0.09, Bregma-0.14, Bregma-0.19, and Bregma-0.24) generated by MERFISH in 3D space. **E** Tissue domain annotations from the previous study (top) and cluster assignments produced by stGCL (bottom). BST: bed nuclei of the strata terminalis; MPA: medial preoptic area; MPN: medial preoptic nucleus; PV: periventricular hypothalamic nucleus; PVH: paraventricular hypothalamic nucleus; PVT: paraventricular nucleus of the thalamus; V3: the third ventricle; and fx: columns of the fornix. **F** Laminar organization of mouse olfactory bulb Stereo-seq data annotated in DAPI-stained image. RMS: rostral migratory stream; ONL: olfactory nerve layer; IPL: internal plexiform layer; GL: glomerular layer; MCL: mitral cell layer; GCL: granule cell layer; EPL: external plexiform layer. **G** Clustering results of STAGATE, GraphST and stGCL. **H** Structural annotation of mouse hippocampus from the Allen Reference Atlas (left) and spatial domains detected by STAGATE, GraphST, and stGCL on mouse hippocampus Slide-seqV2 data (right). The black boxes show the CA1, CA2, CA3, and DG structures identified by stGCL. **I** Layer structure of mouse mPFC STARmap data and Visualization of the spatial domain identified by stGCL. **G** Histology image of PDAC with manual annotations (cancer, duct epithelium, pancreatic and stroma); Clustering results using stGCL embeddings on PDAC data profiled by ST technology. **K** H&E stained image and spatial domains detected by stGCL on breast cancer 10× Xenium data

We first applied stGCL to a human non-small cell lung cancer (NSCLC) dataset captured by NanoString CosMx SMI (high-plex spatial molecular imaging) [35]. This dataset comprises expression profiles and histological profiles with subcellular resolution, containing 20 fields of view (FOVs) and 12 cell type zones (Fig. 4A). In comparison, stGCL portrays cell distribution with higher accuracy and distinctly differentiates the dominant tumor cell-enriched regions and fibroblast-enriched regions, showing consistency that aligns more closely with manual annotation (Fig. 4A-C, Additional file 1: Fig. S5A, C). Subsequently, we utilized the labels obtained from stGCL as grouping criteria and employed CellChat [36] for the discovery of complex ligand-receptor (L-R) interactions between different cell types in NSCLC (Additional file 1: Fig. S5D-G). The results from stGCL corroborate the cell types that frequently communicate in NSCLC, specifically the strong interactions between fibroblasts, endothelial cells, macrophages, T-cells, and tumor cells, as well as within tumor cells themselves (Additional file 1: Fig. S5F, G) [37]. We further visualized the significant L-R pairs involved in the communication between different cell types (Additional file 1: Fig. S5F, G). The results demonstrate that the L-R pair amphiregulin (AREG) and epidermal growth factor receptor (EGFR) expressed within tumors exhibits the strong interaction. Additionally, as previous research has demonstrated, AREG activates the EGFR signaling pathway, which in turn promotes the growth, proliferation, and metastasis of tumor cells [38].

We further evaluated the performance of stGCL using the MERFISH technology which offers single-cell level resolution but detects fewer genes [39]. For the mouse hypothalamic preoptic area MERFISH dataset [39] (transcriptomic modality available), stGCL was able to achieve vertical spatial alignment of five consecutive tissue slices (Bregma-0.04, Bregma-0.09, Bregma-0.14, Bregma-0.19, and Bregma-0.24; Fig. 4D), identifying spatial domain boundaries highly similar to manual annotations (Fig. 4E). In contrast, GraphST and STAGATE failed to separate coherent tissue domains, and SCANPY could not decipher biologically meaningful structural domains other than the third ventricle (V3) region (Fig. 4E and Additional file 1: Fig. S6).

Stereo-seq is an emerging spatial transcriptomics platform that achieves subcellular spatial resolution and generates high-throughput ST datasets on a large number of cells. We evaluated the spatial clustering performance of stGCL using Stereo-seq data (transcriptomic modality available) from mouse olfactory bulb [9] and mouse embryo samples [9]. The laminar structure of the coronal mouse olfactory bulb consists of the olfactory nerve layer (ONL), glomerular layer (GL), external plexiform layer (EPL), mitral cell layer (MCL), internal plexiform layer (IPL), granule cell layer (GCL), and rostral migratory stream (RMS) (Fig. 4F). For qualitative comparison, stGCL accurately identified the outer layer structures of organs (ONL, GL, EPL, and MCL) that matched with annotated layers. In the inner layer structures of the mouse olfactory bulb, it identified clusters (IPL, GCL, and RMS) with a more accurate laminar distribution (Fig. 4G, Additional file 1: Fig. S7A). We further visualized marker genes in specific anatomical regions and discovered a strong correspondence between the clusters detected by stGCL and known marker genes, validating the clustering performance of stGCL (Supplementary Fig. S7B). In addition, we applied stGCL to four mouse embryos datasets acquired at developmental stages E9.5, E10.5, E11.5 and E12.5. stGCL provides intricate representations of tissue organization and organ distribution, closely resembling known anatomical annotations (Additional file 1: Fig. S8). Additionally, stGCL consistently and accurately identified the heart region across all four datasets. These results demonstrate that stGCL is capable of effectively deciphering Stereo-seq data.

Next, we qualitatively tested the performance of stGCL on the Slide-seqV2 dataset of the mouse hippocampus, which contains gene expression profiles with approximate single-cell resolution [40]. We found that stGCL, GraphST and STAGATE were more effective than SCANPY in characterizing spatial domains and demonstrated clearer spatial separation (Fig. 4H and Additional file 1: Fig. S9A). It is noteworthy that stGCL is the only method capable of accurately identifying the "cord-arrow-like" structure in the hippocampal region. The "arrow-like" structure corresponds to the DG region, while the "cord-like" structure corresponds to the CA1, CA2, and CA3 regions of Ammon's horn. The division of CA1, CA2, CA3, and DG clusters in the hippocampal structure by stGCL corresponds to the annotations in the Allen Reference Atlas and has been validated on several known marker genes [27, 41] (Fig. 4H and Additional file 1: Fig. S9B). For example, the secreted synaptic organizer molecule *C1ql2* is a marker gene for dentate gyrus granule cells and is highly expressed in the identified DG domain [42].

Further, we conducted tests on the mouse medial prefrontal cortex (mPFC) STARmap dataset, which features single-cell resolution. This dataset contains transcriptional and histological profiles that are annotated as cortical layers L1, L2/3, L5, and L6 [5]. Compared to the other methods, stGCL achieves the highest ARI and NMI, indicating that the spatial domains identified by stGCL are mostly consistent with the original annotations (Fig. 4I). In addition, the domains detected by stGCL exhibit clear boundaries and low noise (Fig. 4I and Additional file 1: Fig. S10A).

Finally, we verified the generalization ability of stGCL using human pancreatic ductal adenocarcinoma (PDAC) ST data (spot resolution) and human breast cancer 10 × Xenium data (subcellular resolution), both of which include gene expression and histological modalities. stGCL captures PDAC spatial domains that align better with the tissue structure provided by the original study (Fig. 4G and Additional file 1: Fig. S10B). Additionally, stGCL demonstrates spatial domains highly similar to the expected ductal carcinoma in situ (DCIS, named here DCIS #1 and #2), and invasive tumor regions in the breast cancer dataset (Fig. 4K and Additional file 1: Fig. S10C).

All these results demonstrate that stGCL is capable of analyzing spatial transcriptomic data generated on different sequencing platforms, even when these data have varying levels of spatial resolution and different available modalities. By leveraging transcriptional profiles, histological profiles, and spatial information, stGCL can improve the accuracy and reliability of data interpretation, thereby facilitating more precise and informative biological insights.

### stGCL reveals intratumoral spatial heterogeneity from in-house bronchiolar adenoma dataset

**Fig. 5:**
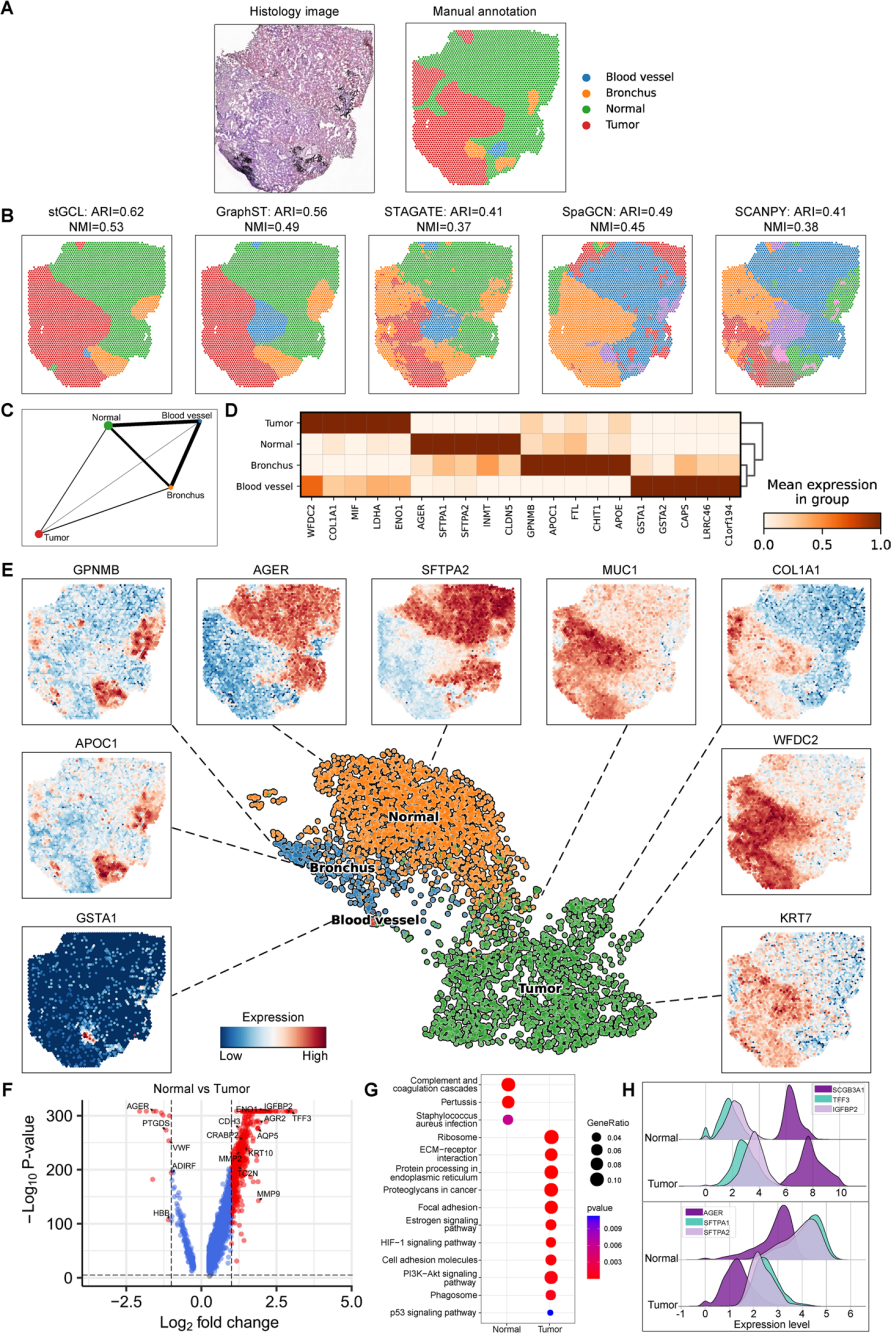
stGCL effectively distinguishes intratumoral spatial heterogeneity in bronchiolar adenoma. **A** H&E stained image and manual annotation of tissue slice by a pathologist. **B.** Clustering results of stGCL, GraphST, STAGATE, SpaGCN and SCANPY. **C**Trajectory inference results using PAGA on stGCL embeddings. **D** Heatmap of the top 5 DEGs for the 4 domains of stGCL. Rows and columns denote domains and genes, respectively. **E** UMAP visualization and scatter plot (Tumor: *MUC1*, *KRT7*, *COL1A1* and *WFDC2*; Normal: *AGER* and *SFTPA2*; Bronchus: *GPNMB* and *APOC1*; Blood vessel: *GSTA1*) generated by stGCL. **F** Volcano plot of DEGs between tumor and normal regions. **G.** KEGG pathways of DEGs between tumor and normal regions. **H** Ridge plots of log-normalized gene expression of top DEGs in tumor and normal regions

Bronchiolar adenoma (BA) is a rare tumor that occurs in the bronchiolar epithelium of the peripheral lung and is known to potentially contain driver gene mutations found in lung cancer [43]. Currently, spatial transcriptomics studies on this type of tumor are relatively scarce. Therefore, we collected fresh tumor tissue for sequencing and engaged professional pathologists to manually segment the regions (see Methods). The pathologist divided the BA tissue into tumor, normal, bronchus and blood vessel regions based on morphological features (Fig. 5A). The domains detected by stGCL are more consistent with manual annotation (ARI = 0.62, NMI = 0.53) and have better regional continuity and less noise (Fig. 5B). For example, stGCL and GraphST display clear spatial patterns, whereas GraphST is unable to identify the tiny tumor region at the top part. The results from STAGATE, SpaGCN and SCANPY are different from manual annotation, with fragmented irregular boundaries between domains. We applied the PAGA algorithm to infer associations between tissue domains and the embedding from stGCL shows closer connections among normal lung tissue, blood vessel and bronchus (Fig. 5C).

To better understand the relationship between the distribution of different tissue regions and their biological functions, we conducted a detailed analysis of differentially expressed genes (DEGs) in the domains identified by stGCL. Initially, we created a heatmap and scatter plot for the DEGs within each region. The results demonstrated that DEGs are well separated in different tissue regions, serving as domain-specific marker genes (Fig. 5D, E). In the tumor region, the *MUC1*, *COL1A1*, *WFDC2* and *KRT7* genes exhibited high expression (Fig. 5E). *MUC1* and *KRT7* are reported to be predefined BA markers, and they are positive in BA [44, 45]. *COL1A1* is a marker of fibroblasts and a key gene in the development and progression of lung cancer and its expression is associated with tumor cell migration, invasion and proliferation [46, 47]. The expression level of *WFDC2* is significantly increased in lung cancer tissues, and *WFDC2* is a potential biomarker for early diagnosis of lung cancer [48]. In addition to the aforementioned results that align with previous studies, stGCL also enabled discoveries of previously unknown cancer-related DEGs. We found that *COL1A1* and *WFDC2* exhibited significant expression differences between normal and tumor tissues, suggesting that they may play a crucial regulatory role in the occurrence and development of BA. Additionally, the results indicate that *AGER* is highly expressed in human lung tissue, particularly in alveolar epithelial cells, and it may play a functional role in epithelial-extracellular matrix interactions [49]. Similar to *AGER*, *SFTPA2* exhibits persistently high expression in the lung, and together with the *SFTPA1*-encoded human surfactant protein A (SP-A), they may play a critical role in lung homeostasis and immunity [50]. For the bronchus region, *GPNMB* encodes a type I transmembrane glycoprotein that is expressed in bronchial epithelial cells [51].

To further reveal the gene expression and functional differences between normal lung tissue regions and tumor tissue regions, we conducted a more detailed differential expression analysis and pathway enrichment analysis for these two areas (Fig. 5F-H). Compared with normal lung tissue regions, some of the highly expressed genes in tumor regions were associated with lung cancer risk (Fig. 5F, H). For example, the matrix metalloproteinase (*MMP*) gene family members *MMP2* and *MMP9* and mesenchymal marker genes play a major role in tumor invasion through proteolysis of the extracellular matrix. They also promote tumor growth and angiogenesis [52]. *SCGB3A1* is related to cell differentiation and proliferation as a lung cancer promoter [53], and *IGFBP2* encodes a pleiotropic oncogenic protein highly expressed in tumor tissues [54]. In addition, *TFF3* is associated with tumor metastasis and prognosis and may serve as a novel biomarker for lung cancer [55]. We further identified that these DEGs are significantly involved in PI3K-Akt signaling pathway, ECM-receptor interaction, Proteoglycans in cancer (Fig. 5G). Most of the DEGs and pathways found in tumor regions are associated with lung cancer, which fits well with the biological insight that BA may contain driver gene mutations found in lung cancer.

The above analysis results demonstrate that using stGCL to analyze spatial transcriptomics data of BA can reveal gene expression differences across different regions of BA tissue, providing a deeper understanding of the spatial heterogeneity of the tumor. Additionally, stGCL identified new potential biomarker target genes, such as *COL1A1* and *WFDC2*. This is significant for exploring the origins and development of BA, identifying key biomarkers and therapeutic targets, and advancing personalized medicine.

## Discussion

Delving deeply into the rich multi-modal information within ST data is crucial for understanding the organization, function and disease progression of heterogeneous tissues. In this work, we proposed stGCL, a cross-modality fusion model based on multi-modal graph contrastive learning. The method is designed to integrate multi-modal data and generate low-dimensional embeddings, thereby enabling spatial domain detection, multi-slice integration, and related downstream analyses. stGCL introduces a novel H-ViT method to learn the histological features of each spot, followed by the adaptive aggregation of transcriptional and histological profiles with spatial neighborhood information via the multi-modal GATE. It further incorporates global information from tissue slices as a guidance signal and utilizes contrastive learning to further enhance joint embeddings of spots. Compared to other methods, stGCL demonstrates strong and consistent performance across different spatial transcriptomics platforms and shows great potential for multi-slice integration.

A key advantage of stGCL lies in the multifunctionality of its latent embeddings. These embeddings not only support accurate spatial domain identification but also enable integration with other advanced bioinformatics algorithms for downstream tasks such as differential expression analysis, intercellular communication, and developmental trajectory inference. For instance, in the analysis of the DLPFC dataset, the combination of stGCL with the PAGA algorithm accurately identified the developmental trajectory from Layer 1 to Layer 6. In the analysis of the bronchiolar adenoma dataset, stGCL identified novel potential biomarkers, offering possibilities for early diagnosis and treatment of the disease. Although these findings are promising, we acknowledge that broader validation across additional datasets and biological contexts will be needed to fully establish the generalizability of the stGCL.

With the rapid advancement of spatial transcriptomics technology, increasing resolution and data throughput also brought new challenges for computational efficiency. We conducted tests on the runtime and GPU memory consumption of stGCL on both real and simulated datasets (Additional file 1: Fig. S11). The test results indicated a direct proportional relationship between the runtime and GPU memory usage with the number of spots. Additionally, incorporating histology image analysis further increases the demands on runtime and GPU memory. Notably, on a breast cancer dataset, generated using the advanced 10 × Xenium technology and containing over 167 k cells, stGCL only required approximately 14 min for processing and consumed about 26.9 GB of GPU memory on a server with Intel(R) Xeon(R) Gold 6258R CPU @ 2.70 GHz and NVIDIA QuADro GV100 GPU (Additional file 1: Fig. S11A). This suggests that stGCL is capable of handling large-scale ST data efficiently.

Although stGCL has demonstrated promising performance, several limitations remain. First, the H-ViT model that stGCL relies on is pre-trained on the ImageNet dataset and has not been specifically fine-tuned for the characteristics of histology images. This may limit its capability to extract spot histological features. Second, while stGCL has been evaluated on diverse ST datasets, its applicability to other spatial omics data types and emerging sequencing technologies remains to be tested. Finally, strategies to better coordinate histologically defined regions with molecularly driven spatial domains are still needed. Addressing these limitations will be an important direction for future work.

## Conclusions

In this study, we introduce stGCL, a versatile cross-modality fusion method based on multi-modal graph contrastive learning, specifically designed for spatial transcriptomics analysis. By systematically integrating gene expression profiles, spatial location, and histological image features, stGCL demonstrated strong performance in spatial domain identification and the reconstruction of 3D tissue structures. Comprehensive benchmarking across diverse datasets shows that stGCL generally outperforms existing methods in the tested scenarios and can scale to large datasets. Taken together, stGCL is poised to become a powerful tool in the field of ST research, offering substantial support for understanding disease progression and identifying key biomarkers.

## Methods

### ST sequencing data preprocessing

We employed the SCANPY package for processing the raw gene expression counts [25]. This process included log-transformation, normalization according to library size, and scaling to achieve unit variance and zero mean. Subsequently, the top 3000 highly variable genes (HVGs) were selected. These HVGs were then used to form the initialized gene expression matrix, which was used as input to stGCL.

### Encoding histology image features using H-ViT

The heterogeneity of biological tissues is reflected in the texture (cell size, shape, and arrangement) and color patterns in histology images. For ST data accompanied by histology images, we proposed a modified ViT [56] model, H-ViT, designed to effectively extract histological features and remove noise in tissue slices (Fig. 1B). Specifically, given a histology image $${\mathbf{\rm I}}_{h}$$, it can be split into $$n$$ spot images based on the size and position of each spot. Next, the image $${\mathbf{I}}_{i}\in {\mathbb{R}}^{h\times w\times c}$$ corresponding to each spot $$i$$ is divided into image patches, where $$\left(h,w\right)$$ denotes the resolution of the spot image and $$c=3$$ denotes the number of channels. We then flatten all patches and assemble them into a $$N\times \left({p}^{2}\times 3\right)$$ sequence $${\mathbf{I}}_{p}$$, where $$\left(p,p\right)$$ represents the resolution of each image patch and $$N=\raisebox{1ex}{$hw$}\!\left/ \!\raisebox{-1ex}{${p}^{2}$}\right.$$ represents the number of patches. In this study, we set $$h=w=40$$ pixels. For each spot image $${\mathbf{I}}_{i}$$, the main steps of the model are as follows:

(1) Patch embedding: To extract the features of each spot image, we employ a simple fully connected layer ${\mathbf{W}}_{p}$ to map the sequence matrix ${\mathbf{I}}_{p}$ to the patch embedding ${\mathbf{E}}_{p}\in {\mathbb{R}}^{N\times 768}$, i.e.,${\mathbf{E}}_{p}={{\boldsymbol{I}}}_{p}{{\boldsymbol{W}}}_{p}$. The obtained embedding matrix is used as the input of the Transformer encoder.

(2) Transformer encoder: The core operation of the Transformer is multi-head self-attention (MSA), which can adaptively learn the attention of patches sequence in the spot image. MSA is a linear combination of $I$ attention heads:

1 $$MSA\left(\mathrm Q,\mathrm K,\mathrm V\right)=\left[head_1,...,head_I\right]{\mathrm W}_{t,}$$

where ${{\boldsymbol{W}}}_{{\boldsymbol{t}}}$ denotes a learnable weight matrix for aggregating attention heads., ${\boldsymbol{Q}}$, ${\boldsymbol{K}}$ and ${\boldsymbol{V}}$ are query, key and value. The attention mechanism can be expressed as:

2 $$head_i=Attention\left(\mathrm{QW}_{\mathrm i}^{\mathrm Q},\mathrm{KW}_{\mathrm i}^{\mathrm K},\mathrm{VW}_{\mathrm i}^{\mathrm V}\right),$$

3 $$Attention\left(\mathrm Q,\mathrm K,\mathrm V\right)=softmax\left(\frac{\mathrm{QK}^T}{\sqrt{d_k}}\right)\mathrm V,$$

where ${{\boldsymbol{W}}}_{i}^{Q}\in {\mathbb{R}}^{768\times 768}$, ${{\boldsymbol{W}}}_{i}^{K}\in {\mathbb{R}}^{768\times 768}$ and ${{\boldsymbol{W}}}_{i}^{V}\in {\mathbb{R}}^{768\times 768}$ refer to learnable weight matrices. In the Eq. (3), the first term $softmax\left({{\boldsymbol{Q}}{\boldsymbol{K}}}^{T}/\sqrt{{d}_{k}}\right)\in {\mathbb{R}}^{N\times N}$ is called the Attention Map, each column of it indicates the attention weights contributed by other elements in the sequence. The second term ${\mathbf{V}}$ denotes the value of the self-attention mechanism. The Transformer encoder is composed of MSA and MLP blocks, and the output is an $N\times 768$ matrix. In the end, H-ViT encodes the histology image into a $n\times \left(N\times 1000\right)$ matrix as the final output. To better capture the histological information and balance it with gene expression information, principal component analysis (PCA) was then employed to extract the first 3000 principal components (PCs) on the output matrix to obtain the histological feature coding matrix $\mathbf{I}$. In each ST data, number of attention heads and MSA layers of the H-ViT model are 12 and 6, respectively.

### Building the spatial neighborhood graph

In ST data, spatial coordinate information can reflect the potential relationship between spots. Therefore, we model the spatial information as an undirected graph $G=\left(V,E\right)$ based on a pre-defined radius$r$. In graph$G$, a node corresponds to a spot, and an edge connects a pair of spots. We use the image coordinates to compute the Euclidean distance between spots, which in turn builds an adjacency matrix $A\in\mathbb{R}^{n\times n}$. $A_{ij}=1$ if the Euclidean distance between spot $i$ and spot $j$ is less than,$r$, otherwise 0. In experiments, we set $r\in \left(d,2d\right)$ ($d$ is the spacing between adjacent spots), which achieves the best performance on most ST data. The self-loop is added to each spot.

### Multi-modal graph contrastive learning for representation enhancement

The proposed stGCL is a cross-modal fusion framework that uses multi-modal graph contrastive learning as its core algorithm, which takes preprocessed gene expression, encoded histological features and constructed spatial neighbor graph as inputs to learn latent spot embeddings (Fig. 1A). The stGCL is structured into three primary phases: 1) Data Augmentation; 2) Multi-modal Graph Attention Auto-Encoder for Structured Embedding; and 3) Contrastive Learning for Enhanced Spot Representations. Detailed descriptions of the implementation for each of these steps are provided subsequently.

#### Data augmentation

Data augmentation, a pivotal technique to amplify sample size and mitigate model overfitting, plays a vital role in graph contrastive learning. In the context of spatial transcriptomics data, this approach lends itself well to the natural modeling of ST data as graph-structured data. Within this framework, each spot node is characterized by two distinct attributes: gene expression and histological features, the latter being included when available. This dual-attribute configuration forms the foundation of our data representation in the proposed framework. We use the corruption function $C$ to generate a negative sample, $\left(\widetilde{\mathbf{X},}\widetilde{\mathbf{I}},\widetilde{\mathbf{A}} \right)=C\left({\boldsymbol{X}},{\boldsymbol{I}},{\boldsymbol{A}}\right)$. In particular, $C$ randomly shuffles the rows of the gene expression matrix and histological feature matrix, and keeps the spatial neighbor graph structure unchanged.

#### Multi-modal graph attention auto-encoder for structured embedding

In this work, we present an extension to the GATE [57] model, leading to the development of the multi-modal GATE. stGCL employs this multi-modal GATE to effectively learn joint latent features. This is achieved by integrating data from different modalities, specifically histological features (when available) and gene expression data. This integration process facilitates a comprehensive understanding and representation of the underlying biological processes, leveraging the strengths of each modality to enrich the learned feature space. Specifically, multi-modal GATE adopts graph attention networks (GAT) [58] as the encoder to learn the spot joint structured embedding $$\mathbf{H}$$ by iteratively aggregating the features of neighbor nodes. The inputs to the encoder are the gene expression matrix $$\mathbf{X}$$, the histological feature matrix $$\mathbf{I}$$, and the adjacency matrix $$\mathbf{A}$$, For each modality, the $$l$$-th encoder layer learns the latent representation of the spot $$i$$$$\left(i\in \left\{\mathrm{1,2},\dots ,n\right\}\right)$$ as follows:

4 $$z_{mi}^{\left(l\right)}=\sigma\left(\sum_{j\in N_i}\alpha_{mij}^{(l)}W_m^{\left(l\right)}z_{mj}^{\left(l-1\right)}\right),m\in\left\{1,2\right\},$$

where ${{\boldsymbol{W}}}_{m}^{\left(l\right)}$ and $\sigma$ represent the learnable weight matrix and nonlinear activation function, respectively. $m$ is the number of modalities, and ${N}_{i}$ is the neighbors of the spot $i$ in the graph$G$. ${z}_{mj}^{\left(l-1\right)}$ is the representation of the spot $j$ generated by the $l-1$ -th encoder layer in the $m$-th modality. ${{\boldsymbol{z}}}_{mi}^{\left(0\right)}$ is initialized as gene expression profile ${\mathbf{x}}_{i}\in {\mathbb{R}}^{{f}_{1}}$ or histological profile${\mathbf{i}}_{i}\in {\mathbb{R}}^{{f}_{2}}$. ${\alpha }_{mij}^{\left(l\right)}$ denotes the normalized attention coefficient output by the $l$-th graph attention layer in the $m$-th modality. We then initialize the joint embedding ${\mathbf{h}}_{i}^{\left(0\right)}\in {\mathbb{R}}^{f}$ by performing a concatenation operation on the latent representations ${\mathbf{z}}_{{1}_{i}}\in {\mathbb{R}}^{{f}_{1}^{\boldsymbol{^{\prime}}}}$ and ${\mathbf{z}}_{2i} \in {\mathbb{R}}^{{f_{2}{\prime} }}$ of each modality, where$={f}_{1}{\prime}+{f}_{2}{\prime}$. The joint structured embedding of spot $i$ is defined as

5 $$h_i^{\left(l\right)}=\sigma\left(\sum_{j\in N_i}\alpha_{ij}^{(l)}\mathrm W^{\left(l\right)}\mathrm h_j^{\left(l-1\right)}\right),$$

where ${\mathbf{h}}_{i}^{\left(l\right)}$ is the embedding of the $l$-th layer. The final output $H\in {\mathbb{R}}^{n\times {f}{\prime}}$ of the encoder is the spot joint embedding, which simultaneously learns gene expression information, histological information and spatial information. ${\alpha }_{ij}^{\left(l\right)}$ represents the normalized attention coefficient output by the $l$-th graph attention layer. The graph attention layer incorporates a self-attention mechanism to dynamically evaluate the relevance between individual spots and their neighboring nodes. Each graph attention layer is computed by

6 $$e_{ij}^{\left(l\right)}=Sigmoid\left(\mathrm v_s^{\left(l\right)^T}\sigma\left(\mathrm W^{\left(l\right)}\mathrm h_i^{\left(l-1\right)}\right)+\mathrm v_k^{\left(l\right)^T}\sigma\left(\mathrm W^{\left(l\right)}\mathrm h_j^{\left(l-1\right)}\right)\right),$$

7 $${\alpha }_{ij}^{\left(l\right)}=\frac{\mathrm{exp}\left({e}_{ij}^{l}\right)}{{\sum }_{0\in {N}_{i}}\mathrm{exp}\left({e}_{io}^{l}\right)},$$

where ${\mathbf{v}}_{s}^{\left(l\right)}$ and ${\mathbf{v}}_{k}^{\left(l\right)}$ denote the trainable parameters. Sigmoid is the sigmoid function. ${e}_{ij}^{\left(l\right)}$ represents the edge weight between the spot $i$ and its neighbor spot $j$ of the $l$-th graph attention layer. Similarly, we can input ${\mathbf{z}}_{mi}^{\left(l-1\right)}$ and ${\mathbf{z}}_{mj}^{\left(l-1\right)}$ to calculate ${\alpha }_{mij}^{\left(l\right)}$ in the graph attention layer. This attention-driven mechanism ensures a more nuanced and context-aware representation of the spatial data, enhancing the overall learning process.    

The decoder part of the multi-modal GATE takes the spot joint embedding $\mathbf{H}$ as input, and each decoder layer reverses the process of its corresponding encoder layer. In this work, the decoder and encoder form a symmetric architecture (Fig. 1A). The spot features of the $l-1$-th layer are reconstructed by the $l$-th layer decoder:

8 $$\widehat h_i^{\left(l-1\right)}=\sigma\left(\sum_{j\in N_i}\widehat\alpha_{ij}^{\left(l\right)}\widehat W^{\left(l\right)}\widehat{\mathrm h}_j^{\left(l\right)}\right).$$

After that, we split the output ${\widehat{\mathbf{h}}}_{i}^{\left(0\right)}\in {\mathbb{R}}^{f}$ into ${\widehat{\mathbf{z}}}_{1i}\in {\mathbb{R}}^{{f}_{1}{\prime}}$ and ${\widehat{\mathbf{z}}}_{2i}\in {\mathbb{R}}^{{f}_{2}{\prime}}$ as the input to the decoder for each modality. Features of gene expression profile and histological profile are reconstructed by the decoder

9 $$\widehat{\mathrm z}_{mi}^{\left(l-1\right)}=\sigma\left(\sum_{j\in N_i}\widehat\alpha_{mij}^{\left(l\right)}\widehat{\mathrm W}_m^{\left(l\right)}\widehat{\mathrm z}_{mj}^{\left(l\right)}\right).$$

stGCL employs half of the trainable parameters (i.e. ${\mathbf{W}}_{m}^{\left(l\right)}={\widehat{\mathbf{W}}}_{m}^{\left(l\right)}$, ${\mathbf{W}}^{\left(l\right)}={\widehat{\mathbf{W}}}^{\left(l\right)}$, ${\alpha }_{m}^{\left(l\right)}={\widehat{\alpha }}_{mi}^{\left(l\right)}$, and ${\alpha }^{\left(l\right)}={\widehat{\alpha }}^{\left(l\right)}$) to avoid overfitting. For gene expression profile and histological profile, we train multi-modal GATE by minimizing the reconstruction loss of spot features:

10 $${L}_{GATE-P}=\sum_{i=1}^{n}\left(\Vert {x}_{i}-{\widehat{z}}_{1i}^{\left(0\right)}\Vert +\Vert {i}_{i}-{\widehat{z}}_{2i}^{\left(0\right)}\Vert \right).$$

For the negative sample, we also use the above multi-modal GATE model to accurately learn the spot joint low-dimensional representation $\widetilde{\mathbf{H}}$. The overall reconstruction loss for multi-modal GATE is summarized as

11 $$\begin{array}{c}L_{GATE}=L_{GATE-P}+L_{GATE-N}\\=\sum_{i=1}^n\left(\Arrowvert x_i-\widehat z_{1i}^{\left(0\right)}\Arrowvert+\Arrowvert i_i-\widehat z_{2i}^{\left(0\right)}\Arrowvert\right)+\sum_{i=1}^n\left(\Arrowvert{\widetilde x}_i-\widetilde z_{1i}^{\left(0\right)}\Arrowvert+\Arrowvert{\widetilde i}_i-\widetilde z_{2i}^{\left(0\right)}\Arrowvert\right),\end{array}$$

where ${\widetilde{{\boldsymbol{z}}}}_{1i}^{\left(0\right)}$ and ${\widetilde{{\boldsymbol{z}}}}_{2i}^{\left(0\right)}$ represent modality-specific features obtained by dividing ${\widetilde{\mathbf{h}}}_{i}^{\left(0\right)}$ into two latent vectors.

#### Contrastive learning for enhanced spot representations

In spatial transcriptomics data derived from the same tissue slice, the gene expression and histological features of individual spots generally align with the global features of the entire tissue slice [59]. This inherent consistency provides a supervised signal that we utilize to guide the learning of spot joint embeddings, thereby enhancing their quality and discriminative power. Specifically, stGCL is designed to maximize the mutual information between each spot's joint embedding and the global summary of the graph. This strategic approach ensures that the learned joint representations capture both local attributes, such as spot-specific expression patterns, and global characteristics, like the overall tissue microenvironment patterns. We employed a readout function $S:{\mathbb{R}}^{n\times {f}{\prime}}\to {\mathbb{R}}^{{f}{\prime}}$ to yield the graph-level summary ${\boldsymbol{s}}=S\left(\mathbf{H}\right)$ by following Velickovic et al. [60]. The negative samples $\left(\widetilde{\mathbf{X}},\widetilde{\mathbf{I}},\widetilde{\mathbf{A}}\right)$ obtained by data augmentation are then fed into the multi-modal GATE to produce the spot joint representation $\widetilde{\mathbf{H}}$. Furthermore, we evaluate the spot embedding-summary pair using the discriminator $D:{\mathbb{R}}^{{f}{\prime}}\times {\mathbb{R}}^{{f}{\prime}}\to {\mathbb{R}}$, and a higher probability score indicates that the spot embedding is more likely to be included in the summary. At the same time, the probability score of positive pair $\left({\mathbf{h}}_{\mathbf{i}},{\boldsymbol{s}}\right)$ is higher than that of negative pair $\left({\widetilde{\mathbf{h}}}_{i},\mathbf{s}\right)$. Finally, the objective function of contrastive learning adopts binary cross-entropy (BCE), which is defined as follows

12 $$L_{CL}=-\frac1{2n}\left(\sum_{i=1}^nE_{\left(\boldsymbol X,\boldsymbol I,\boldsymbol A\right)}\left[\mathrm{log}D\left({\boldsymbol h}_i,\boldsymbol s\right)\right]+\sum_{i=1}^nE_{\left(\widetilde{\boldsymbol X},\widetilde{\boldsymbol I},\widetilde{\boldsymbol A}\right)}\left[\mathrm{log}\left(1-D\left({\widetilde{\boldsymbol h}}_i,\boldsymbol s\right)\right)\right]\right).$$

The overall loss function of stGCL is a combination of multi-modal GATE reconstruction loss and contrastive loss like below:

13 $$L={L}_{GATE}+\beta {L}_{CL},$$

where $\beta$ is the trade-off parameter to control the influence of the contrastive loss term, and its default value is 0.04. In stGCL, the encoder and decoder of the multi-modal GATE module are both a two-layer GAT. We set ${f}_{1}{\prime}$, ${f}_{2}{\prime}$, $f$, ${f}{\prime}$ to 100, 100, 200, 30 respectively, and choose exponential linear unit (ELU) [61] as the activation function. The Adam algorithm [62] is adopted to optimize the objective function $L$ of stGCL. The learning rate, weight decay and training epoch are set to 1e-4, 1e-4 and 1200 (default), respectively. Implementation details are presented in the Additional file 1. For more details, please see Additional file 1: Fig. S12.

### Spatial clustering and visualization

Leveraging the stGCL embeddings $\mathbf{H}$, we employed either the mclust [63] algorithm or the Louvain algorithm [10] to cluster the spots into distinct spatial domains. Each identified cluster represents a spatial domain, encompassing spots that share not only similar expression and histological profiles but also exhibit spatial proximity. In scenarios where the number of spatial domains was predefined, the mclust algorithm was utilized, setting the number of clusters equal to the number of actual labels. In the absence of prior information regarding the ST data, we opted for the Louvain algorithm. We recommend systematically tuning the resolution parameter within a reasonable range (e.g., from 0.2 to 2.0).

Some spots may be erroneously classified into different spatial domains after clustering. To address this, stGCL introduces an optional refinement step. Specifically, for a given spot, stGCL initially examines the spatial clustering outcomes of the spot and its proximal neighbors (typically the 50 nearest neighbors). Following this, stGCL reassigns the spot to the cluster that corresponds to the most frequent label among these neighbors. It is important to note that this refinement step is applied exclusively to datasets where the identification of spatial domains is pertinent and is not recommended for datasets aimed at cell type detection.

For ST datasets with manual annotations, we evaluated clustering performance using the Adjusted Rank Index (ARI) [64] and Normalized Mutual Information (NMI) with higher values indicating superior performance. Additionally, the Kuhn-Munkres [65] algorithm was employed to align cluster labels with ground truth labels effectively. For visualization purposes, the UMAP method was used to project the joint embedding matrix $\mathbf{H}$ into a two-dimensional space, facilitating easier interpretation and analysis of the clustering results.

### Integration of multiple tissue slices via 3D spatial structure reconstruction

The integration of multiple tissue slices offers invaluable insights into the overall structural organization and functional dynamics of the tissue. To capitalize on this, we have enhanced the stGCL method to support the joint analysis of multiple tissue slices. This extension enables a more thorough exploitation of the shared information and spatial context across different slices. Our proposed alignment algorithm is designed to align spatial coordinates from adjacent horizontal slices and consecutive vertical slices, as depicted in Fig. 1C. By aligning these spatial coordinates, we were able to construct a spatial neighborhood graph for multiple slices in a manner analogous to that used for a single slice. Unlike existing methods that often disregard the spatial relationships between tissue slices, our approach fully exploits spatial information to explicitly link multiple slices. This comprehensive graph not only incorporates neighbor information within and across multiple slices but also effectively smoothens spot features on the cut surfaces, thus mitigating batch effects. The gene expression data, histological features, and the spatial neighborhood graph derived from multiple slices are then input into the stGCL framework, following the same processing steps as outlined for single-slice stGCL analysis. The resultant joint embeddings from this analysis of multiple slices are then utilized to identify spatial domains. This process significantly enriches our understanding of tissue organization and functionality, providing a more holistic view than what could be achieved by analyzing single slices in isolation.

To reconstruct the original 3D spatial structure of the tissue, we executed both vertical and horizontal alignments of the slices. Operating under the assumption that the cut surfaces of the slices are nearly identical in shape and size, we developed an alignment algorithm focusing on the edge structure of these surfaces. This method facilitates the integration of multiple slices by aligning their edge structures as closely as possible. For consecutive slices, the cut surface encompasses the entire slice, providing a comprehensive framework for alignment. In the process of vertical integration, we designate a reference tissue slice (referred to as Sect. 1) and then modify the coordinates of the remaining slices to align with this reference. Assuming there are $s$ slices, we calculate the mean of the spot coordinates $\left({x}_{i},{y}_{i}\right)$ located on the edge structures of all slice cut surfaces, thus determining the center spot coordinate $\left({\overline{x} }_{i},{\overline{y} }_{i}\right)$ ($i\in \left\{\mathrm{1,2},...,\left.s\right\}\right.$). Then, we compute the bias between the central spot coordinates of Sect. 1 and those of the other sections ($\Delta {x}_{i}={\overline{x} }_{i}-{\overline{x} }_{1},\Delta {y}_{i}={\overline{y} }_{i}-{\overline{y} }_{1}$). This bias calculation enables us to adjust the positions of the other sections, achieving two-dimensional (2D) alignment across multiple tissue slices. Following the 2D alignment, we proceed to establish the z-axis coordinates for all tissue slices, thereby extending the spatial representation from 2D within each slice to a 3D space encompassing multiple slices. This step is crucial for accurately reconstructing the tissue's original 3D spatial structure and understanding its organization. In particular, the z-axis coordinates of the spots in the $i$-th slice can be defined as

14 $$z_i\left\{\begin{array}{l}0,\;i=1\\\left(i-1\right)\;d+\lambda\sum_{j=1}^{i-1}\;\;\left(l_{j,j+1}\right),i=2,\dots,s\end{array}\right.$$

where $d$ is the thickness of the tissue slice and ${l}_{j,j+1}$ is the separation distance between slice $j$ and slice $j+1$. For instance, the 10× Visium platform generates tissue slices that have a thickness ranging from 10 to 20 μm, which is notably thinner than the diameter of each spot, measured at 55 μm [23]. Given this disparity, it becomes imperative to carefully consider the neighboring spots between consecutive slices, which are constructed based on aligned spatial coordinates and a predefined radius, as illustrated in Fig. 1C.

In the case of horizontal integration, we specifically focused on translating and aligning adjacent tissue slices along the x- and y-axis directions. In this context, the cutting line within a tissue slice is designated as the y-axis, while its perpendicular counterpart is defined as the x-axis. For any given pair of slices, we identify the rightmost spots in the left slice (referred to as Sect. 1) and the leftmost spots in the right slice (Sect. 2), considering these as spots situated on the edge structure of the cutting surface, as depicted by the yellow spots in Fig. 1C. Our initial step involves calculating the mean value ${\overline{y} }_{i}$ ($i\in \left\{1,\left.2\right\}\right.$) of the y-axis coordinates of the spots on the edge structures of both slices. We then determine the maximum value ($\mathrm{max}\left({x}_{1}\right)$) of the x-axis coordinates of all spots in Sect. 1 and the minimum value ($\mathrm{min}\left({x}_{2}\right)$) of the x-axis coordinates of all spots in Sect. 2. Finally, we fix the position of Sect. 1 and adjust the coordinates of Sect. 2 based on the calculated bias ($\Delta x=\mathrm{max}\left({x}_{1}\right)-\mathrm{min}\left({x}_{2}\right),\Delta y={\overline{y} }_{1}-{\overline{y} }_{2}$) to achieve horizontal integration. Furthermore, this approach allows us to treat the aligned slices as a single, unified entity. We can then apply the aforementioned horizontal integration method to align additional slices in various directions.

### Spatial trajectory inference

Trajectory inference represents a significant methodology for deducing the dynamic developmental processes of cells. Utilizing the joint embedding derived through stGCL, we employed the PAGA algorithm [26], sourced from the SCANPY package (‘scanpy.pl.paga_compare’ function) [25], to elucidate the spatial trajectory of these cellular processes.

### Identifying differentially expressed genes

Differentially expressed genes (DEGs) exhibit unique expression patterns across various spatial domains. To identify these DEGs, we performed differential expression analysis utilizing the Wilcoxon test implemented within the SCANPY package. Genes that meet a stringent criterion of a 1% false discovery rate threshold are selected as DEGs, ensuring high confidence in the differential expression results.

### Cell–cell communication

Cell–cell communication is fundamental to cellular function and tissue homeostasis. Leveraging the cluster predictions derived from stGCL embeddings, we utilize the CellChat [36] method to infer ligand-receptor (L-R) interactions among cells.

### Sample collection

Fresh tumor tissue was obtained from a patient diagnosed with primary bronchiolar adenoma (BA) who underwent surgical resection. The patient did not receive any form of neoadjuvant therapy before surgery.

### Spatial transcriptome sequencing

Spatial transcriptome sequencing was conducted using the Visium Technology Platform (10× Genomics). All reagents and consumables were obtained from 10× Genomics, and experiments were performed following the manufacturer’s protocols. Detailed product information is available from 10× Genomics (https://www.10xgenomics.com/products/spatial-gene-expression).

### Manual region segmentation

For the bronchiolar adenoma sample, the pathologist used labelme software [66] to annotate the histology image into distinct regions, each of which can be considered as an irregular polygon. We designed a strategy to identify which region each spot is located in. With spot $i$ as the endpoint, we can make the ray ${l}_{i}$ in any direction. Then we employ the OpenCV package [67] to determine whether the ray passes through the polygon. Which region the spot belongs to can be determined based on the number of times the ray crosses the polygon boundary (crossing number). We can conclude that the spot $i$ is in a polygon $p$ if the crossing number between the ray and polygon $p$ is odd. On the contrary, the spot $i$ is outside polygon $p$ if the crossing number between the ray and polygon $p$ is even. The formula is defined as follows:

15 $$\left\{\begin{array}{l}cn\left(l_i,p\right)\subseteq\Omega_{odd},\;\mathrm{then}\;\mathrm{spot}\;i\;\mathrm{is}\;\mathrm{in}\;\mathrm{the}\;\mathrm{polygon}\;p\\cn\left(l_i,p\right)\subseteq\Omega_{even},\;\mathrm{then}\;\mathrm{spot}\;is\;\mathrm{out}\;\mathrm{the}\;\mathrm{polygon}\;p\end{array}\right.$$

where $cn\left({l}_{i},p\right)$ is the crossing number between the ray ${l}_{i}$ and polygon $p$, and ${\Omega }_{\mathrm{odd}}$ means odd number set, ${\Omega }_{\mathrm{even}}$ means even number set. The proposed manual region segmentation strategy greatly facilitates researchers in automatically converting the annotated information provided by pathologists into label information for each spot. The manual region segmentation strategy we propose significantly aids researchers in the automated conversion of pathologist-provided annotated information into specific label data for each spot. This strategy is pivotal in enabling the transformation of unlabeled datasets into accurately labeled ST data.

### Dataset description

In this study, we utilized 11 ST datasets sourced from diverse platforms, including 10× Visium, STARmap, MERFISH, Slide-seqV2, NanoString CosMx SMI, ST, 10× Xenium, and Stereo-seq, to validate the efficacy of our proposed method. This selection of datasets represents a broad spectrum of technological approaches in the field of spatial transcriptomics, thereby providing a comprehensive basis for assessing the performance and versatility of our method across various platforms.

The dorsolateral prefrontal cortex (DLPFC) 10× Visium dataset is derived from 12 tissue slices of three human brains, each slice comprising 3,460 to 4,789 spots and is manually annotated as DLPFC layers and WM [24].

Mouse brain dataset profiled by 10× Visium is divided into anterior and posterior sections. The mouse anterior brain dataset contains 2,695 spots and 32,285 genes, and is manually partitioned into 52 regions [16].

The mouse olfactory bulb Stereo-seq dataset with sub-cellular level resolution contains 19,109 bins and 14,376 genes [9].

The mouse embryo dataset from the Stereo-seq platform consists of slices (E9.5: 5,913 bins; E10.5: 18,408 bins; E11.5: 30,124 bins; E12.5: 51,365 bins) from four developmental stages [9].

The mouse medial prefrontal cortex (mPFC) STARmap dataset with single-cell resolution maps 1,053 cells with 166 genes and is divided into the four distinct layer structures by the original study [5].

The mouse hypothalamus MERFISH dataset is obtained from tissue slices at Bregma-0.04, −0.09, −0.14, −0.19 and −0.24 mm in the preoptic area of the hypothalamus of animal 1 [39]. Five tissue slices contain 5,488, 5,557, 5,926, 5,803, and 5,543 cells, respectively, and are segmented into eight brain structures including the third ventricle (V3), bed nuclei of the strata terminalis (BST), columns of the fornix (fx), medial preoptic area (MPA), medial preoptic nucleus (MPN), periventricular hypothalamic nucleus (PV), paraventricular hypothalamic nucleus (PVH), and paraventricular nucleus of the thalamus (PVT) [68].

The approximate single-cell resolution mouse hippocampus data obtained from the Slide-seqV2 platform consists of 52,869 spots [40].

The human non-small cell lung cancer (NSCLC) dataset with subcellular level resolution was captured by NanoString CosMx SMI technology [35]. It includes 20 FOVs with a total of 82,843 cells and 12 cell types.

The human pancreatic ductal adenocarcinoma (PDAC) dataset obtained from the ST platform measured 19,738 genes in 428 spots and is manually pathologically annotated into 4 regions [69].

The human breast cancer 10× Xenium dataset contains 167,780 cells with 3 manually annotated tissue regions [70].

The in-house bronchiolar adenoma (BA) dataset profiled by 10× Visium contains 4,002 spots and 36,601 genes, and is manually partitioned into 4 regions. These datasets are summarized in Additional file 1: Table S1.

## Supplementary Information

Additional file 1: Supplementary Figures S1-S15 and Supplementary Tables S1-S2.

Additional file 2: Peer review history.

## Data Availability

The public datasets used in this paper are freely available. (1) Human dorsolateral prefrontal cortex (DLPFC) 10× Visium dataset (http://spatial.libd.org/spatialLIBD/); (2) Mouse brain Sect. 1 10× Visium dataset https://www.10xgenomics.com/resources/datasets/mouse-brain-serial-section-1-sagittal-posterior-1-standard-1-1-0) and mouse brain Sect. 2 10× Visium dataset (https://www.10xgenomics.com/resources/datasets/mouse-brain-serial-section-2-sagittal-anterior-1-standard-1-1-0, (3) Mouse olfactory bulb Stereo-seq dataset (https://github.com/JinmiaoChenLab/SEDR_analysiss); (4) Mouse embryo Stereo-seq dataset (https://db.cngb.org/stomics/mosta/); (5) Mouse medial prefrontal cortex (mPFC) STARmap dataset (https://github.com/zhengli09/BASS-Analysis); (6) Mouse hypothalamus MERFISH dataset (https://datadryad.org/stash/dataset/10.5061/dryad.8t8s248); (7) Mouse hippocampus Slide-seqV2 dataset (https://singlecell.broadinstitute.org/single_cell/study/SCP354/slide-seq-study); (8) Human non-small cell lung cancer (NSCLC) dataset (https://nanostring.com/resources/smi-ffpe-dataset-lung13-data/); (9) Human pancreatic ductal adenocarcinoma (PDAC) ST dataset (https://www.ncbi.nlm.nih.gov/geo/query/acc.cgi?acc=GSM3036911); (10) Human breast cancer 10× Xenium dataset (https://www.10xgenomics.com/products/xenium-in-situ/preview-dataset-human-breast). The in-house dataset used in this study has been deposited on Zenodo, which is publicly accessible. (11) Human bronchiolar adenoma (BA) 10× Visium dataset (https://zenodo.org/record/8185216). The raw sequence data reported in this paper have been deposited in the Genome Sequence Archive [71] in National Genomics Data Center [72], China National Center for Bioinformation/Beijing Institute of Genomics, Chinese Academy of Sciences (accession number: HRA014949) that are publicly accessible at https://ngdc.cncb.ac.cn/gsa-human/browse/HRA014949 [73]. The open-source Python implementation of the stGCL toolkit is available on both GitHub (https://github.com/RuiGaolab/stGCL) [74] and Zenodo (https://zenodo.org/record/8185216) [75]. The source code is released under the MIT license. Detailed manuals and tutorials (https://stgcl-turorials.readthedocs.io/en/latest/) are provided.
