# Supplementary material for: stGCL: a versatile cross-modality fusion method based on multi-modal graph contrastive learning for spatial transcriptomics
Source: Genome Biol. 2026 Jan 28;27:51. doi: 10.1186/s13059-025-03896-w (PMC12924433; doi:10.1186/s13059-025-03896-w)
Supplement: Supplementary file 2 — Additional file 2: Peer review history. [file 13059_2025_3896_MOESM2_ESM.docx]

Review History

**First round of review**

**Reviewer 1**

**Were you able to assess all statistics in the manuscript, including the appropriateness of statistical tests used?**

**Yes:** In my assessment of the manuscript, I thought the statistical analyses and the selection of statistical tests were generally appropriate for the data and research objectives presented. The methodologies used are well-justified, and the interpretation of results aligns with the statistical outcomes.

**Were you able to directly test the methods?** Yes

**Comments to author:**

In this manuscript, Yu et al. proposed a novel method named stGCL, a cross-modal fusion method for spatial domain identification and 3D tissue structure reconstruction. stGCL adopts graph neural networks and contrastive learning to integrate cross-modal features. The authors conducted comprehensive analysis as well as detailed comparison using dataset from different platforms. This manuscript is innovative in concept and substantial in work. However, there are some issues which should be addressed by the authors before publication.

Major:

1. 3D reconstruction represents a significant challenge in current SRT data analysis. stGCL has achieved commendable results in addressing this issue. Please provide detailed insights on how new discoveries or knowledge can be introduced to biology after 3D reconstruction. This is crucial for biologists to interpret biological processes based on the outcomes of stGCL. Additionally, please clarify why samples such as 151673-151676 were chosen for integration in the manuscript, specifically addressing whether there are specific requirements for the selection of slices for 3D reconstruction.

2. I have some concerns about the results on the DLPFC dataset. Maynard et al. used gene-level statistics from both layer-specific versus cell type-specific expression profiles for "spatial registration" in their study. However, the authors did not compare their results with those of Maynard et al. For example, HPCAL1, KRT17, Lam5 and AQP4.

3. I find it interesting that compared to CCST and GraphST, stGCL exhibits significantly better performance, despite all three methods are based on the Deep Graph Infomax model. I would like the authors to clarify what the main differences is compared to these two approaches.

4. For multi-slice integration, several recent computational methods, such as PASTE2 and SPIRAL, are not included in this paper. More discussion is needed to explain the advantages of the proposed method compared to these related methods in the introduction or in the method comparison.

Minor:

1. The authors have provided a detailed analysis workflow and code on ReadTheDocs, but the specific code lacks sufficient comments. Additionally, please provide environment files for stGCL. I spent some time configuring the runtime environment for stGCL. Environment files based on conda or mamba would help bioinformaticians deploy stGCL quickly.

2. For datasets where the number of spatial domains is not known in advance, stGCL utilizes the Louvain algorithm for spatial clustering. Please provide detailed recommendations on selecting the optimal number of spatial domains.

3. To extract histological image features, the authors employed the H-ViT model. Please conduct a comparative analysis with the relevant studies like DeepST, stMVC, and SiGra.

**Reviewer 2**

**Were you able to assess all statistics in the manuscript, including the appropriateness of statistical tests used?**

**Yes: sometimes it is not clear what a replicate was (technical vs. biologic)**

**Were you able to directly test the methods?**

Yes

**Comments to author:**

Yu et al. create a graph contrastive learning-based cross-modality fusion model (stGCL) for using gene expression, spatial information, and histological information for catalouging spatial domains in spatial transcriptomics datasets. They additional present another registration algorithm for 3D reconstruction. They compare their method to some of the current algorithms for classifying spatial transcriptomics spatial areas. While classification of distinct domains are of interest and integrating histological information is of interest, it is unclear how much of an advance that this brings over other existing algorithms where comparisons within the paper and compared to the findings from the other papers themselves are modest and methods are not clear enough to understand justifications, implementation, reproducibility, and assumptions.

Major

* It is claimed that other methods "do not utilize sufficiently the multi-modal information in spatial transcriptomics, and often suffer from low accuracy and robustness in downstream analyses." This is a broad sweeping statement that is not entirely true and is not backed up by data or analyses within the paper.

* The alignment strategy is mentioned as "novel"; however, very few details are shared in the results or methods section for how this is implemented or how it changes from what others have done. Furthermore, there is no data shown within the paper to show that it is better than other state-of-the-art registration methods.

* There are multiple places where comparisons are not quantified and should either quantify them or qualify statements that these are qualitative interpretations (e.g., Figures 3E,F)

* Description of how important parameters of the algorithm (e.g., neighborhood size) and input data parameters (e.g., resolution) affect implementation of the algorithm are not described.

* It is not clear how biological results given could not also be shown by an existing algorithm (e.g., Fig. 5e-h).

Minor

* Comparing to SCANPY is not entirely accurate since there is no spatial information being used and will be localized to the spot/cell/pixel.

* Just measuring ARI is only one parameter out of many that could be used to compare accuracy in domain assignment

* There is no discussion of how different algorithms could be better or worse at recognizing different types of spatial domains, where there can't be one algorithm that will recognize all because there are different biological questions across different spatial domains.

* It is not clear what is being generated whether cell type or regions in Fig. 4A-C

Aligning regions such as Fig. 5A with pathologist is naturally biasing towards histological region identification rather than molecular driven region identification and should be acknowledged the trade-offs that are inherent in this.

**Authors Response**

**Point-by-point responses to the reviewers’ comments:**

**Response to the reviewers**

**Submission ID**: GBIO-D-23-01839

**Title**: stGCL: A versatile cross-modality fusion method based on multi-modal graph contrastive learning for spatial transcriptomics

Dear Reviewers,

We sincerely thank you for the great efforts to deal with our manuscript as well as the constructive and thoughtful feedback. These comments encouraged us to substantially improve the quality of our work. According to your comments and suggestions, we have revised our manuscript (highlighted in red) and source code thoroughly. Please find our itemized response to each reviewer’s concerns in below.

Thank you once again for the valuable feedback on our work.

Rui Gao *et al.*

School of Control Science and Engineering

Shandong University

Jinan 250061, P.R. China

**Response to Reviewer #1**

**Comments to Author:**

In this manuscript, Yu et al. proposed a novel method named stGCL, a cross-modal fusion method for spatial domain identification and 3D tissue structure reconstruction. stGCL adopts graph neural networks and contrastive learning to integrate cross-modal features. The authors conducted comprehensive analysis as well as detailed comparison using dataset from different platforms. This manuscript is innovative in concept and substantial in work. However, there are some issues which should be addressed by the authors before publication.

**Response:** Thank you sincerely for your appreciation of the significance of our work and your insightful comments. We have carefully considered each of your suggestions and have incorporated them into our manuscript. We believe that these revisions have significantly improved the quality and clarity of our paper. Below are our detailed responses to each of your valuable comments.

**Major:**

1. 3D reconstruction represents a significant challenge in current SRT data analysis. stGCL has achieved commendable results in addressing this issue. Please provide detailed insights on how new discoveries or knowledge can be introduced to biology after 3D reconstruction. This is crucial for biologists to interpret biological processes based on the outcomes of stGCL. Additionally, please clarify why samples such as 151673-151676 were chosen for integration in the manuscript, specifically addressing whether there are specific requirements for the selection of slices for 3D reconstruction.

**Response:** Thank you for raising this critical issue. We appreciate the reviewer’s insightful comments and concerns on the 3D reconstruction capability of the stGCL toolkit, and apologize for any confusion caused by our previous description.

Generally, most spatial omics are measured on 2-dimensional (2D) tissue slices, but molecular dynamics and intercellular actions in the tissues can be rarely embodied on them, which is critical information in understanding developmental biology and heterogenous tumor microenvironment (TME). By aligning consecutive 2D sections, stGCL constructs a three-dimensional (3D) tissue profile of the entire tissue, providing a bird’s-eye view that enables a more comprehensive understanding of the tissue ecosystem. For example, on four DLPFC slices (151673-151676) generated with 10x Visium, stGCL’s multi-slice integrated analysis achieved higher clustering accuracy than the analysis using only one slice and produced the white-matter (WM) structure that more closely matched the expected anatomy (Fig. 3D and Additional file 1: Fig. S1). This demonstrates that incorporating cross-slice information substantially improves spatial tissue structure identification.

We selected slices 151673-151676 because they constitute a widely used and highly representative benchmark dataset: four consecutive, homogeneous slices from the same tissue, each with well-curated ground-truth annotations—ideal conditions for a clean assessment of stGCL’s 3D reconstruction performance. Beyond this benchmark, stGCL also successfully rebuilt 3D tissue architecture (i) across 12 DLPFC slices obtained from three adult donors and (ii) across two sagittal anterior mouse brain slices that exhibited evident batch effects, aligning all slices accurately within a common 3D coordinate system (Additional file 1: Fig. S2). In summary, stGCL is able to robustly integrate multiple slices generated on the same platform to produce coherent 3D reconstructions. These clarifications have been incorporated into the Results section titled "stGCL improves integration of multiple tissue slices" of the revised manuscript.

2. I have some concerns about the results on the DLPFC dataset. Maynard et al. used gene-level statistics from both layer-specific versus cell type-specific expression profiles for "spatial registration" in their study. However, the authors did not compare their results with those of Maynard et al. For example, HPCAL1, KRT17, Lam5 and AQP4.

**Response:** Thank you for your insightful comments. Following your suggestion, we have compared the stGCL results with the manually annotated layers reported by Maynard et al. for the human DLPFC dataset. Specifically, we analyzed several well-established layer-specific markers, including HPCAL1 (L2), MOBP (WM), LAMP5 (L2/3), and AQP4 (L1). We note that the marker referred to by the reviewer as "Lam5" should correctly be written as LAMP5 (Lysosomal-Associated Membrane Protein 5).

Taking DLPFC slice 151674 as an example, we examined the expression patterns of these layer-specific markers within the annotated cortical layers and compared them with clusters identified by stGCL (Figure R1). The clusters closely matched their corresponding cortical layers. These results further demonstrate the capability of stGCL to accurately capture tissue architectures. We have included these results in Fig. S13 of the Additional file 1.

Figure R1. Violin plots showing expression of four layer-specific marker genes across manually annotated layers (top) and stGCL-inferred clusters (bottom).

3. I find it interesting that compared to CCST and GraphST, stGCL exhibits significantly better performance, despite all three methods are based on the Deep Graph Infomax model. I would like the authors to clarify what the main differences is compared to these two approaches.

**Response:** Thank you for raising this important methodological concern regarding the differences among stGCL, CCST, and GraphST. Although stGCL, CCST, and GraphST are all inspired by the Deep Graph Infomax (DGI) framework, stGCL differs substantially in several key methodological aspects, contributing to its superior performance. These differences can be summarized into three main areas: 1) cross-modal integration, 2) spatial-aware contrastive learning, and 3) 3D tissue reconstruction capability.

1) stGCL explicitly integrates multi-modal information (spatial transcriptomics and histological data) through a unified, spatial-graph-based contrastive learning framework. In contrast, CCST and GraphST primarily rely on single-modal data (spatial transcriptomics). By effectively leveraging complementary molecular and morphological features, stGCL achieves higher accuracy in spatial domain identification.

2) Unlike CCST and GraphST, stGCL employs a novel spatial-aware graph contrastive loss that explicitly incorporates spatial adjacency and modality interactions. Specifically, it integrates histological features with spatial transcriptomic information into its contrastive learning loss, significantly enhancing sensitivity to spatially coherent molecular patterns. This design encourages learning spatially meaningful embeddings.

3) CCST operates solely within individual slices, performing only 2D spatial domain identification. While GraphST supports vertical integration across multiple slices by aligning spatial coordinates with the PASTE algorithm and constructing a joint neighborhood graph, its analysis fundamentally remains a joint analysis within a unified 2D spatial coordinate system. Specifically, GraphST does not explicitly define spatial coordinates along the vertical dimension (z-axis), nor does it reconstruct a true continuous 3D spatial structure. In contrast, stGCL explicitly incorporates spatial coordinates along the z-axis and constructs a genuine three-dimensional spatial graph. Furthermore, stGCL explicitly integrates multi-modal features during vertical integration, enabling more precise and biologically coherent 3D reconstructions.

These distinctions have been included in Background section of the revised manuscript.

4. For multi-slice integration, several recent computational methods, such as PASTE2 and SPIRAL, are not included in this paper. More discussion is needed to explain the advantages of the proposed method compared to these related methods in the introduction or in the method comparison.

**Response:** We thank the reviewer to point this out. Following your kind suggestion, we have introduced the PASTE2 and SPIRAL methods in the revised manuscript and further elaborated on the advantages of stGCL in comparison to these methods.

Briefly, PASTE2 achieves slice alignment in the presence of partially overlapping slices by solving a partial fused Gromov-Wasserstein optimal transport problem. This method incorporates a model selection procedure to estimate the fraction of overlap between slices and can optionally utilize information derived from histological images. SPIRAL is a computational framework designed to integrate and align spatial transcriptomic data from different experiments, conditions, and technologies. It consists of two modules: SPIRAL-integration, which integrates datasets via graph domain adaptation, and SPIRAL-alignment, which aligns spatial coordinates using a clustering-based optimal transport strategy. In comparison, stGCL integrates transcriptomics and histological imaging features simultaneously within a graph contrastive learning framework. It explicitly incorporates spatial neighborhood and cross-modality consistency into the loss function, enabling more precise spatial domain identification and accurate 3D tissue reconstruction. Unlike SPIRAL, which aligns slices based primarily on spatial proximity and transcriptional similarity without using histological imaging data, stGCL comprehensively integrates both imaging and gene expression modalities. Moreover, although PASTE2 also utilizes histological image information, stGCL applies a spatial-aware contrastive learning approach to more effectively leverage complementary multi-modal information for reconstructing biological structures.

Additionally, stGCL employs an H-ViT model to extract deep image-derived features from histological data, providing stronger alignment and identification capabilities compared to the simplistic RGB mean used by PASTE2. While PASTE2 employs imaging information primarily as an auxiliary alignment aid, stGCL integrates image and gene expression data deeply through advanced deep learning methods, thus delivering a more powerful multi-modal integration capacity. Furthermore, PASTE2 primarily functions as a spatial alignment tool and does not directly address downstream tasks like domain identification, whereas stGCL represents a comprehensive analytical framework capable of slice alignment, spatial domain identification, and 3D reconstruction simultaneously.

Lastly, both PASTE2 and SPIRAL focus mainly on two-dimensional spatial alignment without explicitly defining the vertical (z-axis) coordinate. In contrast, stGCL explicitly incorporates vertical spatial coordinates, facilitating precise 3D tissue reconstruction and enhancing biological interpretability. We have updated these descriptions accordingly in the Background section of the revised manuscript.

**Minor:**

1. The authors have provided a detailed analysis workflow and code on ReadTheDocs, but the specific code lacks sufficient comments. Additionally, please provide environment files for stGCL. I spent some time configuring the runtime environment for stGCL. Environment files based on conda or mamba would help bioinformaticians deploy stGCL quickly.

**Response:** Thank you for your valuable suggestion. We have carefully reviewed and enhanced the comments within the provided code, ensuring improved clarity and readability to facilitate users' understanding of the analysis workflow. In addition, as recommended, we have prepared a ready-to-use runtime environment file (environment.yml) based on conda to simplify the deployment of stGCL. This environment file has been added to our GitHub repository (https://github.com/RuiGaolab/stGCL). Bioinformaticians can rapidly configure and replicate our computational environment using a single command (conda env create -f environment.yml -n stGCL).

2. For datasets where the number of spatial domains is not known in advance, stGCL utilizes the Louvain algorithm for spatial clustering. Please provide detailed recommendations on selecting the optimal number of spatial domains.

**Response:** Thank you very much for raising this question. The Louvain algorithm is a community detection method that automatically identifies communities (here, spatial domains) by optimizing network modularity. Thus, it does not require users to specify the number of clusters in advance. However, it includes a resolution parameter that controls the granularity of clustering results. A lower resolution yields fewer clusters (coarser domains), while a higher resolution produces more clusters (finer domains).

We recommend systematically tuning the resolution parameter within a reasonable range (e.g., from 0.2 to 2.0, as described in the revised manuscript) and evaluating the stability and biological interpretability of the resulting domains. Additionally, users can incorporate prior biological knowledge—such as known anatomical structures, cell types, or marker gene expression patterns—to interpret clustering outcomes and guide the optimal number of spatial domains. Furthermore, visualization of the hierarchical trees and UMAP clustering results can also assist in determining an optimal domain number. We have included a detailed description of spatial clustering using the Louvain algorithm in the "Spatial clustering and visualization" section of the revised manuscript.

3. To extract histological image features, the authors employed the H-ViT model. Please conduct a comparative analysis with the relevant studies like DeepST, stMVC, and SiGra.

**Response:** Thank you for your valuable feedback. Following your insightful suggestion, we conducted a comparative analysis between our H-ViT-based histological feature extraction method and those used in related studies (DeepST, stMVC, and SiGra) in the revised manuscript.

DeepST utilizes the Inception v3 model to extract spot-level histological image features. Although this CNN-based method effectively captures local morphological features, its convolutional structure has limited capability in modeling long-range spatial dependencies within histological images.

stMVC employs a ResNet-50 model to extract histological image features. Similarly, its convolution-based architecture predominantly focuses on local and intermediate-scale features, limiting the ability to effectively capture global spatial contexts.

SiGra directly flattens cropped multi-channel spot images into one-dimensional vectors as the initial image features. While this approach is straightforward, it cannot effectively capture essential image texture features such as cell size, shape, and spatial arrangements, resulting in suboptimal feature representation.

In comparison, our proposed stGCL employs the H-ViT model, specifically designed to simultaneously capture local image details and global spatial context from histological images. The transformer-based structure of H-ViT effectively models global spatial relationships, generating more informative, comprehensive, and biologically meaningful embeddings, thereby significantly improving downstream tasks, including spatial domain identification and multi-modal integration.

To demonstrate this further, we conducted an empirical comparison of these four image feature extraction methods using the DLPFC slice 151674 dataset. Figure R2 shows that the proposed H-ViT-based approach achieves higher spatial domain identification accuracy, aligning well with our previous explanations. These results have been included in Fig. S14 of the Additional file 1.

Figure R2. Performance comparison of spatial domain identification using four histological image feature extraction methods: stGCL (H-ViT), stMVC (ResNet-50), DeepST (Inception v3), and SiGra (flattened multi-channel images).

**Response to Reviewer #2**

**Comments to Author:**

Yu et al. create a graph contrastive learning-based cross-modality fusion model (stGCL) for using gene expression, spatial information, and histological information for catalouging spatial domains in spatial transcriptomics datasets. They additional present another registration algorithm for 3D reconstruction. They compare their method to some of the current algorithms for classifying spatial transcriptomics spatial areas. While classification of distinct domains are of interest and integrating histological information is of interest, it is unclear how much of an advance that this brings over other existing algorithms where comparisons within the paper and compared to the findings from the other papers themselves are modest and methods are not clear enough to understand justifications, implementation, reproducibility, and assumptions.

**Response:** We would like to express our gratitude to the reviewer for taking the time to review our manuscript, and appreciate your valuable feedback and insightful comments, which have helped us to improve the clarity and impact of our work.

Novelty and advancement:

We would like to emphasize that while there are existing tools for spatial transcriptomics (ST) analysis, stGCL offers several unique contributions to the field. Firstly, stGCL systematically encodes and integrates gene expression data, spatial information, and histological features via a multi-modal graph contrastive learning framework. And we developed a novel H-ViT method to effectively encode histological features. stGCL introduces a novel strategy for spatial coordinate correction and registration specifically tailored for multi-slice integration. stGCL integrates two common tasks in ST—spatial domain identification and 3D tissue structure reconstruction—into a comprehensive toolkit, providing researchers with a streamlined and efficient workflow. Secondly, the benchmarking results presented in our revised manuscript demonstrate the advantages of stGCL over existing tools in terms of accuracy and robustness. To ensure fair and comprehensive evaluation, comparisons were performed across 10 publicly available datasets spanning multiple platforms and resolutions, as well as validated on an in-house bronchiolar adenoma dataset. stGCL consistently outperformed existing methods in both single-slice and multi-slice analyses across various performance metrics, highlighting its superiority and practicality. Specifically, stGCL improved spatial domain identification and provided more accurate 3D tissue structure reconstructions, thereby enabling researchers to explore deeper biological relationships and tissue heterogeneity mechanisms.

Implementation and reproducibility:

We acknowledge the concerns raised regarding the implementation and reproducibility of stGCL. To address these concerns comprehensively, we have enhanced the methodological descriptions within our manuscript. To ensure that stGCL is user-friendly and accessible to the broader scientific community, we provide detailed documentation and step-by-step guidelines for stGCL installation, setup, and execution (https://stgcl-turorials.readthedocs.io/en/latest/).

**Major:**

1. It is claimed that other methods "do not utilize sufficiently the multi-modal information in spatial transcriptomics, and often suffer from low accuracy and robustness in downstream analyses." This is a broad sweeping statement that is not entirely true and is not backed up by data or analyses within the paper.

**Response:** Thank you for your insightful comment regarding the overgeneralized statement in our manuscript. We acknowledge that the original wording was indeed too broad and lacked sufficient precision. In response, we have carefully revised this part of the Background to better reflect our intended meaning. Specifically, we updated the sentence as follows: "However, these methods typically emphasize local spatial contexts or gene expression profiles alone and may not fully leverage complementary multi-modal information (particularly high-resolution texture features derived from histology images), potentially limiting their performance in predicting tissue structures [1, 2]."

Furthermore, the ablation study presented in our manuscript demonstrates the importance of histological image features: when histological information is disregarded (w/o H-ViT), the performance of stGCL decreases by 6.7% (Fig. 2E). This finding highlights that multi-modal integration of histology images with transcriptomic profiles significantly contributes to the accurate delineation of spatial domains.

2. The alignment strategy is mentioned as "novel"; however, very few details are shared in the results or methods section for how this is implemented or how it changes from what others have done. Furthermore, there is no data shown within the paper to show that it is better than other state-of-the-art registration methods.

**Response:** Thanks for raising this important comment, and we sincerely apologize for any confusion caused by the unclear description. Specifically, in the "Integration of multiple tissue slices via 3D spatial structure reconstruction" subsection within the Methods section, we provide detailed implementation steps of alignment approach, clearly outlining both vertical and horizontal integration. We introduce a novel alignment algorithm based on the edge structures of the cut surfaces of tissue slices, enabling effective correction of spatial coordinates across multiple slices. By aligning these coordinates, we construct a comprehensive spatial neighborhood graph for multiple slices—similar to our single-slice approach—capturing neighbor information both within and across slices and effectively smoothing spot features at the cut surfaces to mitigate batch effects. Gene expression data, histological features, and this spatial neighborhood graph derived from multiple slices are then input into the stGCL framework, following the same processing steps as described for single-slice analysis.

Moreover, we enhanced the Background and Methods sections of the revised manuscript to clearly highlight the motivation behind our alignment strategy and its distinct advantages compared to other state-of-the-art methods:

• SpaGCN jointly analyzes multiple serial slices by creating a block adjacency matrix and concatenating expression matrices, but does not correct spatial locations between slices.

• STAGATE requires pre-aligned slices as input and cannot handle horizontal integration of multiple adjacent slices.

• GraphST utilizes the PASTE algorithm to align spatial coordinates in two dimensions only, thus limiting accurate 3D reconstruction.

The alignment of spatial coordinates across tissue slices remained highly challenging because of the differences in the structure of the slices and their position on the array. Therefore, the effective and precise integration of spatial data, whether horizontal or vertical, is crucial in ST research.

In contrast, our proposed stGCL implements a novel alignment algorithm specifically designed to register spatial coordinates based on the edge structure of tissue slices. This strategy efficiently integrates spatial transcriptomics data across multiple slices (both vertically and horizontally) and achieves precise, comprehensive 3D reconstruction. Unlike existing methods that often disregard the spatial relationships between tissue sections, our approach fully exploits spatial information to explicitly link multiple slices. By clearly defining the z axis, we achieve precise three-dimensional reconstructions of tissue architecture. Furthermore, the integration of transcriptomic and histological data enhances spatial domain identification across slices.

To further demonstrate the superior performance of our method, we conducted comparative analyses in the "stGCL improves integration of multiple tissue slices" subsection of the Results section:

• Vertical integration: We compared stGCL with SCANPY, SEDR, and STAGATE, demonstrating that stGCL achieves higher clustering accuracy and superior vertical integration performance (Fig. 3A-D).

• Horizontal integration: Compared with SpaGCN and GraphST, stGCL more accurately identifies shared layered structures across slices (Fig. 3E, F and Additional file 1: Fig. S3). Additionally, visualization of known marker genes (Additional file 1: Fig. S4) further validated the biological relevance of the spatial domains identified by stGCL across multiple slices.

These results have been included in the "Integration of multiple tissue slices via 3D spatial structure reconstruction," "stGCL improves integration of multiple tissue slices," and Background sections, clearly demonstrating the novelty, implementation details, and comparative advantages of our alignment strategy.

3. There are multiple places where comparisons are not quantified and should either quantify them or qualify statements that these are qualitative interpretations (e.g., Figures 3E, F)

**Response:** Thank you for bringing this important point to our attention. As recommended, we have clarified in the revised manuscript that the comparisons presented in Figures 3E-F are qualitative interpretations. Specifically, due to the absence of ground-truth labels in the mouse brain dataset, we compared the clear structures identified by different methods with those from the Allen reference atlas. We further visualized marker genes in specific anatomical regions and discovered a strong correspondence between the clusters detected by stGCL and known marker genes, validating the clustering performance of stGCL (Additional file 1: Fig. S4). Moreover, we have indicated that similar qualitative comparisons have been applied to other datasets throughout the manuscript that also lack ground-truth labels. We have updated the corresponding Results section in the revised manuscript.

4. Description of how important parameters of the algorithm (e.g., neighborhood size) and input data parameters (e.g., resolution) affect implementation of the algorithm are not described.

**Response:** Thank you very much for highlighting this point. We are sorry for the confusion. We have systematically evaluated the effects of key hyperparameters in stGCL, as shown in Additional file 1: Fig. S12. Additionally, in the "Building the spatial neighborhood graph" subsection, we have clearly described how stGCL constructs the spatial neighborhood graph based on a pre-defined radius $r$ and provided guidance on setting this radius. Following your kind suggestion, we further assessed how variations in the neighborhood radius $r$ impact the performance of stGCL. Figure R3 demonstrates that optimal performance on the DLPFC slice 151674 dataset is achieved when $r$ ranges from $d$ to $2d$ (where $d$ is the spacing between adjacent spots). For this particular dataset (DLPFC slice 151674), $d$ is approximately 137 pixels, and our experiments confirmed that the highest Adjusted Rand Index (ARI) value was achieved at $r=150$ pixels.

Moreover, in the "stGCL demonstrates robustness and scalability on spatial transcriptomic data across platforms and resolutions" subsection, we conducted extensive benchmarking with existing methods on multiple datasets, including both single-cell level and spot-level spatial data generated by different platforms. These experiments consistently highlight stGCL’s superior performance in spatial domain identification.

Figure R3. Clustering accuracy of stGCL with different neighborhood radius $r$ on DLPFC slice 151674.

5. It is not clear how biological results given could not also be shown by an existing algorithm (e.g., Fig. 5e-h).

**Response:** We greatly appreciate the reviewer’s thoughtful comment and apologize for any confusion caused by our previous description. To further validate the performance of stGCL, we performed comparative analyses with representative existing methods on our in-house bronchiolar adenoma (BA) dataset, as described in the "stGCL reveals intratumoral spatial heterogeneity from in-house bronchiolar adenoma dataset" subsection. The spatial domains identified by stGCL not only match manual annotations more closely than those produced by other approaches, but also display better regional continuity and less noise.

Importantly, to gain a deeper understanding of the relationship between the spatial distribution of tissue regions identified by stGCL and their biological functions, we conducted detailed differential expression and pathway enrichment analyses (Fig. 5E-H). Thus, these panels showcase the biological insights derived from the spatial clustering results of stGCL. Overall, stGCL can disentangle gene expression differences across distinct regions of BA and significantly advances our understanding of intratumoral spatial heterogeneity. In addition, stGCL discovered novel biomarkers and potential therapeutic targets.

**Minor:**

1. Comparing to SCANPY is not entirely accurate since there is no spatial information being used and will be localized to the spot/cell/pixel.

**Response:** Thank you for your valuable feedback. At present, the methods to identify spatial domains could be mainly divided into two categories, non-spatial and spatial clustering methods [3]. Traditional non-spatial clustering algorithms, such as Louvain clustering-based Scanpy method, only take gene expression data as input, and the resulting clusters may not be contiguous due to the lack of consideration of spatial information and histology [4]. In our analysis, SCANPY was specifically included as a representative baseline method for non-spatial clustering. This comparison highlights the significance and effectiveness of integrating spatial information and multi-modal data, as accomplished by stGCL.

2. Just measuring ARI is only one parameter out of many that could be used to compare accuracy in domain assignment.

**Response:** Thank you for your valuable suggestion regarding clustering evaluation metrics. In response, we have incorporated the Normalized Mutual Information (NMI) clustering metric to more comprehensively evaluate the reliability of stGCL. We have updated the experimental results accordingly in the revised manuscript and Additional file 1.

3. There is no discussion of how different algorithms could be better or worse at recognizing different types of spatial domains, where there can't be one algorithm that will recognize all because there are different biological questions across different spatial domains.

**Response:** Thank you for your constructive comment. We agree that no single method can consistently achieve optimal performance across all types of spatial domains. For instance, stGCL has distinct advantages over GraphST and STAGATE in sequencing-based spatial transcriptomics techniques that include histological images, primarily due to stGCL's effective encoding of histological features. Moreover, if capturing fine-grained tissue structures is needed, stGCL and GraphST may outperform other methods because both leverage contrastive learning frameworks that simultaneously model local properties and global structural patterns.

In Additional file 1: Table S1, we summarize all datasets used in our manuscript, indicating that stGCL is designed as a versatile cross-modality fusion method for accurate and robust integration of gene expression, spatial location, and histological features. In the subsection "stGCL demonstrates robustness and scalability on spatial transcriptomic data across platforms and resolutions", we demonstrate that stGCL consistently outperforms existing methods across multiple mainstream spatial transcriptomics platforms. This superior performance is primarily attributed to the deep fusion of transcriptional, spatial, and histological information, generating embeddings with both rich representational capacity and strong discriminative power. However, we acknowledge that stGCL's performance in spatial domain identification remains uncertain for other types of spatial omics data or future novel sequencing technologies. We intend to continuously refine our algorithm as new data types become available. These results have been included in Discussion section of the revised manuscript.

4. It is not clear what is being generated whether cell type or regions in Fig. 4A-C.

**Response:** Thank you for raising this important point. We apologize for any confusion caused by our description of Fig. 4A-C. To clarify, the regions shown in Fig. 4A-C represent spatial domains enriched for specific cell types, consistent with findings reported in [5]. We have clarified this point in the revised manuscript.

5. Aligning regions such as Fig. 5A with pathologist is naturally biasing towards histological region identification rather than molecular driven region identification and should be acknowledged the trade-offs that are inherent in this.

**Response:** Thank you for your insightful comment. We agree that directly comparing algorithm-identified regions with pathologist-defined annotations (as shown in Fig. 5A) may inherently bias the results toward histologically-defined rather than purely molecularly-driven spatial domains. Indeed, many existing algorithms treat regions defined by pathologists (based on histological images) as the ground truth, and hyperparameters are often adjusted to match these annotations closely, resulting in a natural preference for morphologically distinct and clearly delineated regions. This approach enhances histological interpretability but may result in certain spatial patterns based on molecular-level differences not being clearly identified.

Our proposed method, stGCL, partially mitigates this issue because we consistently use the same hyperparameters across datasets from the same sequencing platform, rather than specifically tuning hyperparameters to fit the annotations in Fig. 5A. Thus, spatial domains identified by stGCL inherently integrate both morphological and molecular features, balancing histological interpretability and sensitivity to molecular heterogeneity. We acknowledge this inherent limitation and discuss the need for future approaches to better address this trade-off in Discussion section of the revised manuscript.

**References**

[1] Xu C, Jin X, Wei S, et al. DeepST: identifying spatial domains in spatial transcriptomics by deep learning. Nucleic Acids Research, 2022, 50(22): e131.

[2] Zuo C, Zhang Y, Cao C, et al. Elucidating tumor heterogeneity from spatially resolved transcriptomics data by multi-view graph collaborative learning. Nature Communications, 2022, 13(1): 5962.

[3] Long Y, Ang K S, Li M, et al. Spatially informed clustering, integration, and deconvolution of spatial transcriptomics with GraphST. Nature Communications, 2023, 14(1): 1155.

[4] Hu J, Li X, Coleman K, et al. SpaGCN: Integrating gene expression, spatial location and histology to identify spatial domains and spatially variable genes by graph convolutional network. Nature methods, 2021, 18(11): 1342-1351.

[5] Tang Z, Li Z, Hou T, et al. SiGra: single-cell spatial elucidation through an image-augmented graph transformer. Nature Communications, 2023, 14(1): 5618.

**Second round of review**

**Reviewer 1**

The authors have addressed my concerns, and I think the revisions have significantly improved the quality of the manuscript. At this stage, I have a few minor points which should be addressed before the manuscript is ready for submission.
1. The English is generally clear and sufficient for understanding the manuscript. However, some proofreading would help improve the polish and overall presentation. For example, the phrases “techniques measures the spatial distribution” and “the sample are often dissected” contain grammatical errors. “The stGCL adopts modified H-ViT” should be “The stGCL adopts a modified H-ViT”.
2. The clusters in Figure 4H start from 1, but in Figure S9A they start from 0. Same issue can be found in Fig.S8 and Fig.S9. Please standardize the cluster numbering across figures to avoid confusion.
3. In Fig.3C, the cluster labels are not aligned. The labels in Fig.4C are slightly misaligned. The cluster labels in Fig.S3 is duplicated.
4. Some abbreviations in the manuscript are labeled multiple times, such as 'white matter (WM)'.
5. The authors provided the codes for the datasets used in this study on ReadtheDocs, but the code lacks key comments, resulting in poor readability.
6. In the attached figures, some labels are bolded while one is not, for example in Fig. S12. Please ensure consistency.

**Reviewer 2**

The reviewers should be commended for their work in revising the manuscript based on my prior comments. While the manuscript has been significantly improved, there are still concerns in the quantitative evaluation and comparison of the developed method.

1. While there is some indication of alignment quality (Fig. 3A-B), this is not convincing enough to make the claims regarding substantial improvements over other methods. More comparisons, metrics, and data should be shown to support these claims. Similarly, analysis of 3D data should be done to show that improvements impact the downstream analysis to validate the level of impact claimed in the paper.

2. Conclusions in general across the paper are still strong despite the limited number of comparisons/situations. These should be tempered as well as highlighting limitations in the Discussion/Conclusion.

**Authors Response**

**Point-by-point responses to the reviewers’ comments:**

We are very grateful to the two reviewers for their constructive comments, which definitely helped us improve our paper greatly. We have made revisions in the manuscript (highlighted in red) that reflect the insights and suggestions. Below is the point-by-point response to all the reviewers’ comments.

====================================================

**Response to Reviewer #1**

**Comments to Author:**

The authors have addressed my concerns, and I think the revisions have significantly improved the quality of the manuscript. At this stage, I have a few minor points which should be addressed before the manuscript is ready for submission.

**Response:** We thank the reviewer for acknowledging our efforts to address the concerns. Below, we address each of the main points raised.

1. The English is generally clear and sufficient for understanding the manuscript. However, some proofreading would help improve the polish and overall presentation. For example, the phrases "techniques measures the spatial distribution" and "the sample are often dissected" contain grammatical errors. "The stGCL adopts modified H-ViT" should be "The stGCL adopts a modified H-ViT".

**Response:** Thank you for pointing these out. We have carefully proofread the entire manuscript and revised the expressions to improve clarity and grammar. Specifically:

• We corrected “techniques measures the spatial distribution” to “techniques measure the spatial distribution”.

• We revised “the sample are often dissected” to “the samples are often dissected”.

• We changed “The stGCL adopts modified H-ViT” to “The stGCL adopts a modified H-ViT”.

We have also checked the rest of the manuscript and polished the language to improve readability. Thank you again for all your careful reading.

2. The clusters in Figure 4H start from 1, but in Figure S9A they start from 0. Same issue can be found in Fig.S8 and Fig.S9. Please standardize the cluster numbering across figures to avoid confusion.

**Response:** Thank you very much for your careful observation. We agree with the reviewer that the inconsistent cluster numbering across figures may cause confusion. To address this, we have standardized the cluster numbering throughout all relevant figures, including Fig. 4H, Fig. S8, and Fig. S9, so that the clusters are consistently labeled starting from 1.

3. In Fig.3C, the cluster labels are not aligned. The labels in Fig.4C are slightly misaligned. The cluster labels in Fig.S3 is duplicated.

**Response:** Thank you for raising these issues with the figure labeling. We have carefully re-checked the figures and corrected the problems as follows: the cluster labels in Fig. 3C have been realigned, the slight misalignment in Fig. 4C has been fixed, and the duplicated labels in Fig. S3 have been removed.

4. Some abbreviations in the manuscript are labeled multiple times, such as 'white matter (WM)'.

**Response:** We thank the reviewer to point this out. Following your kind suggestion, we have thoroughly reviewed the manuscript and removed redundant definitions of abbreviations such as white matter (WM).

5. The authors provided the codes for the datasets used in this study on ReadtheDocs, but the code lacks key comments, resulting in poor readability.

**Response:** Thank you for your valuable suggestion. We have added key comments to the released code to improve its readability and facilitate its use by other researchers.

6. In the attached figures, some labels are bolded while one is not, for example in Fig. S12. Please ensure consistency.

**Response:** Thank you very much for raising this question. We have revised the figures and ensured that all labels, including those in Fig. S12, are consistently formatted.

**Response to Reviewer #2**

**Comments to Author:**

The reviewers should be commended for their work in revising the manuscript based on my prior comments. While the manuscript has been significantly improved, there are still concerns in the quantitative evaluation and comparison of the developed method.

**Response:** We appreciate the reviewer’s encouraging comments regarding our manuscript. Based on your valuable feedback, we have made several substantial revisions to address your concerns and further strengthen our manuscript:

1. While there is some indication of alignment quality (Fig. 3A-B), this is not convincing enough to make the claims regarding substantial improvements over other methods. More comparisons, metrics, and data should be shown to support these claims. Similarly, analysis of 3D data should be done to show that improvements impact the downstream analysis to validate the level of impact claimed in the paper.

**Response:** We appreciate the reviewer’s insightful comments and concerns on the alignment quality of the stGCL. Following your valuable suggestion, we have conducted additional experiments to systematically evaluate alignment performance. Figure R1 visualizes alignment results of five hypothalamic preoptic area MERFISH slices (Bregma-0.04, Bregma-0.09, Bregma-0.14, Bregma-0.19, and Bregma-0.24). The slices are colored according to whether each spatial region is correctly aligned (“Region Match”) or misaligned (“Region Mismatch”), where spots are shown in green if correctly matched to the expert annotation and in red if mismatched. We also report quantitative alignment accuracy (acc) for each method, using SANTO [1] and PASTE [2] as advanced baselines for comparison. As shown in Figure R1, our proposed stGCL consistently achieves higher accuracy across all slices (average acc = 0.73), demonstrating substantial improvements in alignment quality compared with Raw data, SANTO, and PASTE. The corresponding implementation has been made available in our GitHub repository (https://stgcl-turorials.readthedocs.io/en/latest/).

In addition, we performed 3D data analysis on the aligned slices. Since SANTO and PASTE are designed solely for alignment and cannot be directly applied to downstream analyses, we instead compared stGCL with representative multi-slice integration methods, including SCANPY, STAGATE, and GraphST (the latter using PASTE for inter-slice spatial coordinate alignment). Using spatial domain identification as a representative downstream task, stGCL achieved higher ARI and NMI scores and demonstrated superior integration performance (Figure R2). We have included these results in Fig. 4D-E as well as in Figs. S6 and S15 of the Additional file 1.

Figure R1. Visualization of alignment results of different methods on the five hypothalamic preoptic area MERFISH slices (Bregma-0.04, Bregma-0.09, Bregma-0.14, Bregma-0.19, and Bregma-0.24). Slices are colored according to alignment correctness of spatial region.

Figure R2. Clustering results obtained by stGCL, GraphST, STAGATE and SCANPY methods on the mouse hypothalamic preoptic area MERFISH dataset.

2. Conclusions in general across the paper are still strong despite the limited number of comparisons/situations. These should be tempered as well as highlighting limitations in the Discussion/Conclusion.

**Response:** Thank you for bringing this important point to our attention. We agree that the conclusions of our study should be stated more cautiously given the limited number of comparisons and application scenarios. As recommended, we have carefully revised the Discussion and Conclusion sections to temper the strength of our statements. Specifically, we now emphasize that while our proposed stGCL method demonstrates promising performance, the findings are based on a limited set of datasets and tasks. We explicitly discuss potential limitations, including the reliance on an ImageNet-pretrained backbone for histology feature extraction, the need for validation on additional datasets and emerging spatial omics technologies, and the challenge of aligning histologically defined regions with molecularly derived domains. We believe these revisions provide a more balanced perspective while acknowledging the contributions and limitations of the present work.

**References**

[1] Li H, Lin Y, He W, et al. SANTO: a coarse-to-fine alignment and stitching method for spatial omics. Nature Communications, 2024, 15(1): 6048.

[2] Zeira R, Land M, Strzalkowski A, et al. Alignment and integration of spatial transcriptomics data. Nature Methods, 2022, 19(5): 567-575.

**Second round of review**

**Reviewer 1**

The authors have addressed my previous comments. I recommend the manuscript for publication in its current form.

**Reviewer 2**

The authors have addressed my concerns.
